# Systemic Inflammation and Adverse Outcomes in Patients With Atherosclerotic Cardiovascular Disease and Chronic Kidney Disease

**DOI:** 10.1016/j.jacadv.2026.102765

**Published:** 2026-04-22

**Authors:** Alexander T. Sandhu, Adam Furst, Fatima Rodriguez, Neil Kalwani, David Maron, Shriram Nallamshetty, Natasha Din, Kimberly Zitko, Kat Khachatourian, Jeffrey R. Skaar, Ivy Tonnu-Mihara

**Affiliations:** a Division of Cardiology, Department of Medicine, University of California Los Angeles, Los Angeles, California, USA; b Greater Los Angeles Veterans Affairs Healthcare System, Los Angeles, California, USA; c Palo Alto Veterans Affairs Healthcare System, Palo Alto, California, USA; d Division of Cardiology, Stanford University School of Medicine, Stanford, California, USA; e Stanford Prevention Research Center, Stanford University School of Medicine, Stanford, California, USA; f Novo Nordisk Inc., Plainsboro, New Jersey, USA

**Keywords:** chronic renal insufficiency, epidemiology, health resources, inflammation, coronary artery disease

## Abstract

**Background:**

Systemic inflammation (SI), measured by high-sensitivity C-reactive protein (hsCRP), is common in both atherosclerotic cardiovascular disease (ASCVD) and chronic kidney disease (CKD).

**Objectives:**

The objectives of the study was to evaluate the association between SI and cardiovascular and economic outcomes among Veterans with ASCVD and CKD.

**Methods:**

We conducted an analysis of Veterans with ASCVD and CKD with hsCRP testing between 2008 and 2022. We classified Veterans as with SI (hsCRP 2-10 mg/L) and without SI (hsCRP <2 mg/L). The primary outcome was the 3-point major adverse cardiovascular event (MACE) composite: death, myocardial infarction, and stroke. Additional outcomes included acute care utilization and health care costs. We evaluated the adjusted association between SI and clinical outcomes, acute care utilization, and costs via Cox regression, negative binomial regression, and gamma regression, respectively.

**Results:**

Veterans with SI (n = 39,006) were younger (72.5 [SD: 9.1] vs 73.6 [SD: 9.1] years; *P* < 0.001) and had a higher prevalence of peripheral arterial disease (48.4% [18,860/39,006] vs 43.8% [10,152/23,176]; *P* < 0.001). Over a median follow-up of 4.2 years (IQR: 2.1-7.0; total of 307,074 person-years), the MACE rate was 11.64 (95% CI: 11.48-11.79) compared with 8.66 (95%CI: 8.49-8.83) per 100 patient-years in the group with vs without SI (*P* < 0.001). After adjustment, the HR for SI was 1.40 (95% CI: 1.36-1.43) for MACE. SI was significantly associated with increased hospitalizations (IRR: 1.24; 95% CI: 1.20-1.29) and $4,115 (95% CI: $1,878-$6,353) in additional costs over 1 year.

**Conclusions:**

Among Veterans with ASCVD and CKD, SI was associated with an increased risk of MACE and higher health care utilization and costs.

Both atherosclerotic cardiovascular disease (ASCVD) and chronic kidney disease (CKD) are major public health burdens in the United States, affecting 18 and 35 million adults, respectively.^1^^,^^2^ The relationship between these 2 chronic diseases is increasingly recognized with the unifying framework of cardiovascular-kidney-metabolic disease.^3^ The 2 conditions not only share common risk factors, including hypertension, diabetes, and obesity, but individuals with both conditions also experience worse clinical outcomes.^4^^,^^5^

Systemic inflammation (SI), as assessed by biomarkers such as high-sensitivity C-reactive protein (hsCRP), is commonly observed among individuals with comorbid ASCVD and CKD.^6^ Multiple prospective studies have demonstrated that SI is associated with an increased risk of major adverse cardiovascular events (MACE).^7^, ^8^, ^9^, ^10^ However, there are limited real-world data regarding the clinical and economic outcomes of individuals with comorbid ASCVD and CKD given limited measurement of hsCRP during routine care.^6^

The Veterans Health Administration (VHA), the nation’s largest integrated health system, provides a unique opportunity to better understand the role of SI among Veterans with comorbid ASCVD and CKD. We evaluated the association between SI and cardiovascular and renal clinical and economic outcomes among Veterans with hsCRP testing between 2008 and 2022.

## Methods

### Data

We used electronic health record and administrative data from VHA’s Corporate Data Warehouse in addition to claims for Veterans’ purchased care and Medicare fee-for-service from 2005 through 2023. The VHA Corporate Data Warehouse includes demographics, outpatient and inpatient encounters, diagnoses, procedures, laboratory results, vital signs, and pharmacy dispensing records. Purchased care and Medicare claims data were used to identify clinical care and medication prescriptions provided to Veterans outside of the VHA health care system. Mortality data were based on the VHA Mortality Data Repository and the National Death Index. Underlying cause of death was available via National Death Index data through 2022.

### Study population and high-sensitivity C-reactive protein

We identified Veterans with ASCVD and CKD with an hsCRP measurement between 2008 and 2022. We first identified hsCRP testing using laboratory codes (Logical Observation Identifiers, Names, and Codes; Supplemental Table 1). We restricted the analysis to the first hsCRP test performed in the outpatient setting in which the Veteran was between 18 and 89 years old and had established ASCVD and CKD at the time of testing. Because hsCRP testing and results were analyzed retrospectively, we were unable to determine the clinical indication of testing. ASCVD was identified based on International Classification of Diseases-9/10 diagnosis codes or coronary or peripheral arterial revascularization procedure codes (Supplemental Table 1) within the 3 years before the hsCRP test. We included CKD stage 3 or 4 (estimated glomerular filtration rate [eGFR] 15 to 60 mL/min/1.73 m^2^ based on the CKD-EPI formula)^11^ or CKD stage 2 with urinary albumin-to-creatinine ratio ≥200 mg/g due to the increased risk associated with albuminuria.^12^ The eGFR and urinary albumin-to-creatinine ratio were based on the most recent tests within 3 years pre-hsCRP. For individuals with missing creatinine levels, we also identified CKD stages 3 or 4 based on International Classification of Diseases diagnosis codes (see Supplemental Table 1). The date of the hsCRP test was set as the index date.

To identify a cohort with active VHA care, we excluded hsCRP tests without at least 1 diagnosis and clinical encounter in the preceding year. We also excluded tests in which the Veterans had active comorbidities in the preceding year that cause severe SI and increase the risk of adverse clinical outcome and health care costs: cancer receiving chemotherapy, metastatic cancer, HIV, or chronic osteomyelitis (Supplemental Table 1). Given much of the clinical morbidity and economic cost is noncardiovascular, we excluded these conditions to provide a more conservative estimate of the association between hsCRP and clinical and economic outcomes. In addition, we excluded tests within 60 days of myocardial infarction (MI) or stroke hospitalization given the transient rise in hsCRP levels following these acute events. In a sensitivity analysis, we also excluded tests among Veterans with established systemic autoimmune disease.

For the primary analysis, we defined SI as hsCRP levels ≥2 mg/L and ≤10 mg/L. Prior analyses have used levels ≥2 mg/L as a benchmark for inflammatory risk.^10^^,^^13^ As has been performed previously, we set an upper threshold of hsCRP ≤10 mg/L because marked elevation of hsCRP may be related to acute infections or other major systemic disorders with increased associated clinical morbidity and cost.^14^, ^15^, ^16^

We identified a second contemporary cohort with ASCVD and CKD without hsCRP test (non-hsCRP cohort) to compare patient characteristics between those with and without hsCRP test. Using an index date of January 1, 2023, we identified Veterans with ASCVD and CKD without hsCRP testing during a 3-year lookback period based on the same methods as described previously.

### Study variables

We captured patient sociodemographics, vital signs, comorbidities, laboratory values, and medications. Race and ethnicity classifications were based on patient self-reporting. Vital signs were based on the most recent values in the 3 years before the index date. Based on each patient’s home address, we determined their Center for Disease Control Social Vulnerability Index, a measure of community risk at the census tract level.^17^

We identified comorbidities based on International Classification of Diseases-9/10 diagnoses (Supplemental Table 1). We identified a surrogate for frailty based on the presence of at least 2 diagnoses previously used to capture frailty using claims data.^18^ We captured cardiovascular procedures using Current Procedural Terminology and International Classification of Diseases procedure codes (Supplemental Table 1). Laboratory measurements were based on standardized laboratory codes (Logical Observation Identifiers, Names, and Codes). We identified the use of outpatient medications for ASCVD and CKD based on prescription fills in the VHA or Medicare Part D data in addition to non-VHA medication entries in the electronic health record.

### Clinical Outcomes

Our primary clinical outcome was a 3-point MACE composite: all-cause death, MI, or stroke. MI and stroke were both defined as an acute care hospitalization with a principal diagnosis of MI and stroke, respectively.^19^^,^^20^ Secondary clinical outcomes included a 5-point MACE composite: all-cause death, MI, stroke, coronary revascularization, or peripheral arterial revascularization; each individual component of the composite outcome; and cardiovascular death. Revascularization was identified based on procedural codes (Supplemental Table 1). Cardiovascular death was defined based on an underlying cause of death International Classification of Diseases code for cardiovascular disease (I.00-I.99) (Supplemental Table 1).^21^^,^^22^

### Acute care utilization and health care costs

For acute care utilization, we captured the frequency of all-cause hospitalizations, cardiovascular hospitalizations, and emergency department visits without hospitalization. We identified cardiovascular hospitalizations based on the principal diagnosis with the same diagnosis codes used for identifying cardiovascular death. We identified emergency department visits based on the VA stop codes—a coding system used by the VHA that identifies location of services, and place of service codes for non-VHA care.

For health care costs, we analyzed the following cost categories: total, inpatient, outpatient, pharmacy, and cardiovascular-related. Cardiovascular-related costs were identified based on an associated International Classification of Diseases code consistent with cardiovascular disease (Supplemental Table 1). VHA costs were based on average cost methods in which services are weighted by Medicare relative value weights and adjusted to the total cost of care within the VHA.^23^ For Medicare and community care cost data, we used the total amount paid, including patient out-of-pocket costs. All costs were inflated to 2023 U.S. dollars via the Personal Health Care Index.^24^ We evaluated health care resource utilization and costs over multiple time periods postindex date: 6 months, 1 year, 3 years, and 5 years.

### Statistical analysis

We first described patient characteristics via mean and SD for continuous variables and count and percentages for categorical variables, comparing characteristics between those with and without SI and those with hsCRP >10 vs those with hsCRP ≥2 m/L and ≤10 mg/L. We also compared patient characteristics between those with and without hsCRP testing using standardized mean differences (SMDs) via Cohen d and Mahalanobis distance.

For clinical outcomes, we compared incidence rates of each clinical outcome between those with and without SI. As a sensitivity analysis, we repeated the analysis while including those with hsCRP >10 mg/L in the SI cohort. We also evaluated the adjusted association between SI and outcomes using Cox survival models. We assessed the proportional hazards assumption using Schoenfeld residuals. Given the global test demonstrated nonproportional hazards, the HRs are therefore interpreted as time-averaged estimates, as has been suggested.^25^ We censored events on the date of last known encounter. For nonfatal events, we also censored outcomes on the date of death, as previously suggested for etiological analyses.^26^ For cardiovascular death, we censored outcomes on December 31, 2022, given the lack of cause of death data for 2023. Given the potential for nonlinearity of association between outcomes and hsCRP level, we repeated these analyses with alternate modeling of hsCRP. First, we modeled hsCRP via quartiles, with the lowest quartile as the reference category. Second, we modeled hsCRP as a restricted cubic spline with 4 knots (based on 5th, 35th, 65th, and 95th percentiles) and graphically displayed the results.

We modeled the association between SI and health care resource utilization using negative binomial regression models, with an offset to account for varying follow-up time, and displayed the results via incidence rate ratios. We modeled the association between SI and health care costs via gamma regression models, with the results displayed as the average marginal cost associated with SI. Given inpatient and cardiovascular costs are often zero, we modeled these cost categories via two-stage models with a logit model for predicting any health care costs and the gamma model for predictions conditional on cost being greater than zero. We excluded individuals who died or reached end of follow-up before completing the designated follow-up period.

We evaluated 2 nested adjusted models for all outcomes: (model A) adjusted for age, sex, and year of hsCRP measurement and (model B) additionally adjusted for the Charlson Comorbidity Index (CCI), prior MI, prior coronary revascularization, smoking status, body mass index, eGFR, and medications known to improve outcomes among patients with ASCVD and CKD (lipid-lowering therapies, antiplatelets, anticoagulants, angiotensin-converting enzyme inhibitors/angiotensin receptor blockers, sodium-glucose cotransporter-2 inhibitors, and glucagon-like peptide-1 receptor agonists). These variables were selected a priori due to their probable associations with the outcomes and hsCRP levels and their established clinical relevance. In a sensitivity analysis, we repeated the primary analysis with adjustment for all characteristics with significant imbalance across groups at baseline. All continuous variables were modeled as a restricted cubic splines (based on 5th, 35th, 65th, and 95th percentiles) with 4 knots to account for nonlinearity.

We evaluated missingness patterns among model covariates. As detailed in the Supplemental Methods, missingness for body mass index and eGFR were both below 2%. There was not missingness for other model covariates. We found the missingness of body mass index and eGFR were not strongly associated with other observed clinical characteristics. Given the low rate of missingness, we used mean imputation; we also included missing indicators to account for nonrandom missingness.^27^ Furthermore, we performed a complete case analysis of the primary clinical outcome.

We evaluated the association between SI and outcomes while adjusting for age [in nonage subgroup], sex, and year of hsCRP across key clinical subgroups: age <65 vs ≥65, ASCVD type (coronary artery disease [CAD] vs peripheral arterial disease vs cerebrovascular disease vs polyvascular disease), CKD stages (stage 2 with urinary albumin-to-creatinine ratio ≥200 mg/g vs stage 3 vs stage 4), diabetes mellitus, heart failure, and CCI score ≤ median vs > median). We did not include subgroup interactions by sex given the substantial imbalance in proportion and characteristics between male and female sex in our cohort, as is standard in most VA patient populations. We evaluated multiplicative interaction between the SI and the subgroup characteristic using interaction terms in the Cox models to identify whether HRs differed significantly across subgroups.

We used a threshold of *P* ≤ 0.05 for statistical significance. Data for this project are not publicly available, but code can be made available to VA investigators on request. The content is solely the responsibility of the authors and does not necessarily represent the official views of VA. This study was approved by the Stanford University Institutional Review Board and follows the Strengthening the Reporting of Observational Studies in Epidemiology (STROBE) reporting guidelines.

## Results

### Baseline characteristics

**Figure 1:**
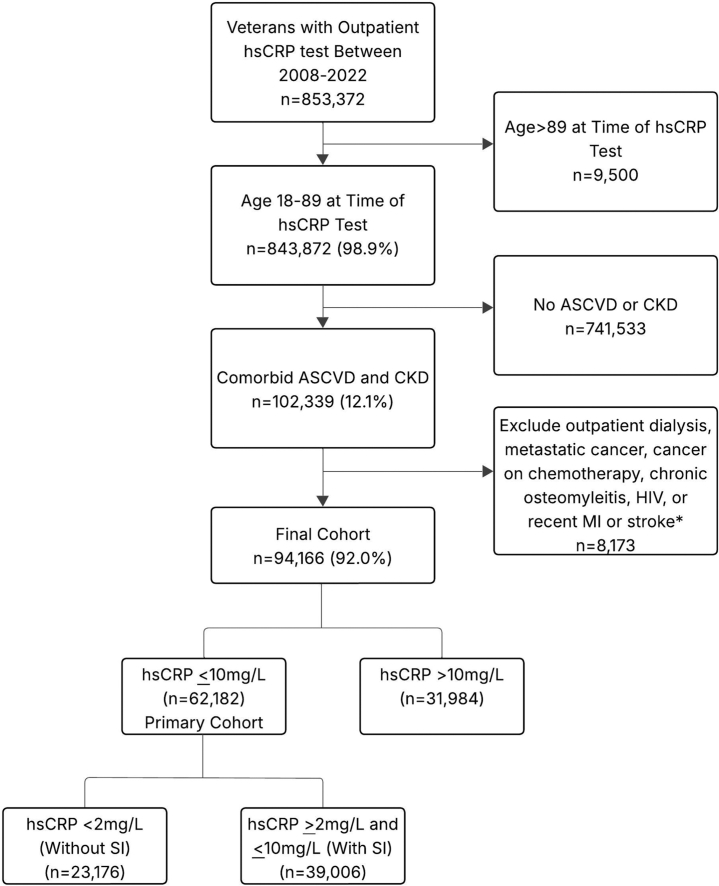
**CONSORT Diagram** ∗Excluding individuals with outpatient dialysis, metastatic cancer diagnosis, cancer chemotherapy, osteomyelitis, or HIV within the prior year and individuals with MI or stroke hospitalization within the prior 60 days. ASCVD = atherosclerotic cardiovascular disease; CKD = chronic kidney disease; hsCRP = high-sensitivity C-reactive protein; MI = myocardial infarction; SI = systemic inflammation.

We identified 102,339 Veterans with ASCVD and CKD with outpatient hsCRP testing between 2008 and 2022 (Figure 1). After applying additional clinical exclusion criteria described previously, there were 94,166 Veterans (92.0%). Supplemental Table 2 compares characteristics of Veterans excluded due to the clinical exclusion criteria. Furthermore, we compared Veterans in our cohort to a contemporary cohort of Veterans with ASCVD and CKD without hsCRP testing (Supplemental Table 3). We found our study cohort was more likely to have cerebrovascular disease (32.9% [30,983/94,166] with hsCRP testing vs 18.1% [47,034/259,901] without hsCRP testing; SMD = 0.345) and rheumatoid arthritis (7.7% with hsCRP testing [7,225/94,166] vs 2.7% [6,987/259,901] without hsCRP testing; SMD = 0.226). All other characteristics were similar between groups with SMDs under 0.2.

For the primary analysis, we excluded the 34.0% (31,984/94,166) Veterans with hsCRP >10 mg/L. Individuals with hsCRP >10 m/L had similar rates of systemic autoimmune conditions but were more likely to be frail (32.6% [10,414/31,984] vs 23.3% [9,094/39,006]; *P* < 0.001) and had greater overall comorbidity (median CCI score of 5 [IQR: 3-7] vs 4 [IQR: 2-6], *P* < 0.001) compared to those with hsCRP ≥2 and ≤10 mg/L (Supplemental Table 4). The primary analysis was restricted to the 62,182 Veterans with hsCRP ≤10 mg/L, with 39,006 Veterans (62.7%) with SI (hsCRP ≥2 mg/L), and 23,176 Veterans (37.3%) without SI (hsCRP <2 mg/L). Supplemental Figure 1 displays the full distribution of hsCRP levels.

Table 1 represents the baseline characteristics of patients with and without SI. Veterans with SI were younger than those without SI (72.5 [SD: 9.1] vs 73.6 [SD: 9.1]; *P* < 0.001); the proportion with female sex was similar in both groups (4.1% [1,609/39,006] vs 4.0% [920/23,176], *P* = 0.35). Most Veterans with and without SI had CAD (75.0% [29,247/39,006] vs 75.7% (17,534/23,176]; *P* = 0.06). Veterans with SI were more likely to have peripheral arterial disease (48.4% [18,860/39,006] vs 43.8% [10,152/23,176]; *P* < 0.001) and heart failure (34.7% [13,547/23,176] vs 27.8% [6,449/23,176]; *P* < 0.001). The mean eGFR was lower among those with SI (49.0 mL/min/1.73 m^2^ [SD: 12.6] vs 49.8 mL/min/1.73 m^2^ [SD: 11.9]; *P* < 0.001). Veterans with SI were more likely to have rheumatoid arthritis (8.0% [3,134/39,006] vs 7.1% [1,641/23,176]; *P* < 0.001).

**Table 1:** Baseline Characteristics of Veterans With and Without Systemic Inflammation

	Overall (N = 62,182)	hsCRP ≥2 and ≤10 mg/L (n = 39,006)	hsCRP <2 mg/L (n = 23,176)	*P* Value
Demographics				
Age, years, mean (SD)	73.0 (9.1)	72.5 (9.1)	73.6 (9.1)	<0.001
Female, n (%)	2,529 (4.1)	1,609 (4.1)	920 (4.0)	0.354
Race, n (%)				
Asian	442 (0.7)	186 (0.5)	256 (1.1)	<0.001
American Indian/Native American	542 (0.9)	345 (0.9)	197 (0.9)	
Black	11,401 (18.3)	7,178 (18.4)	4,223 (18.2)	
Pacific Islander/Native Hawaiian	594 (1.0)	337 (0.9)	257 (1.1)	
White	44,686 (71.9)	28,198 (72.3)	16,488 (71.1)	
Missing	4,517 (7.3)	2,762 (7.1)	1755 (7.6)	
Ethnicity, n (%)				
Non-Hispanic/Latinx	55,212 (88.8)	34,713 (89.0)	20,499 (88.4)	0.059
Hispanic/Latinx	4,056 (6.5)	2,521 (6.5)	1,535 (6.6)	
Missing	2,914 (4.7)	1772 (4.5)	1,142 (4.9)	
Rural designation, n (%)	14,540 (23.4)	9,279 (23.8)	5,261 (22.7)	<0.001
Drive time to a primary care, minutes, median (IQR)	27.58 (23.78)	27.72 (24.24)	27.35 (22.99)	0.058
CDC SVI by quartile, n (%)				
<0.25	10,471 (16.8)	6,251 (16.0)	4,220 (18.2)	<0.001
0.25-<0.50	14,200 (22.8)	8,878 (22.8)	5,322 (23.0)	
0.50-<0.75	15,352 (24.7)	9,583 (24.6)	5,769 (24.9)	
0.75-1.00	15,344 (24.7)	9,803 (25.1)	5,541 (23.9)	
Missing	6,815 (11.0)	4,491 (11.5)	2,324 (10.0)	
Comorbidities				
Atherosclerotic cardiovascular disease				
Coronary artery disease	46,781 (75.2)	29,247 (75.0)	17,534 (75.7)	0.061
Cerebrovascular disease (i.e. stroke and TIA)	20,623 (33.2)	12,630 (32.4)	7,993 (34.5)	<0.001
Peripheral artery disease	29,012 (46.7)	18,860 (48.4)	10,152 (43.8)	<0.001
Myocardial infarction	3,292 (5.3)	1907 (4.9)	1,385 (6.0)	<0.001
Revascularization, coronary or peripheral arterial	8,881 (14.3)	5,662 (14.5)	3,219 (13.9)	0.032
Other cardiovascular disease				
Atrial fibrillation/atrial flutter	15,001 (24.1)	10,041 (25.7)	4,960 (21.4)	<0.001
Heart failure	19,996 (32.2)	13,547 (34.7)	6,449 (27.8)	<0.001
Valvular heart disease	13,424 (21.6)	8,473 (21.7)	4,951 (21.4)	0.296
Ventricular arrhythmias	4,414 (7.1)	2,870 (7.4)	1,544 (6.7)	0.001
Systemic autoimmune disorders				
Myositis	120 (0.2)	91 (0.2)	29 (0.1)	0.004
Multiple sclerosis	231 (0.4)	143 (0.4)	88 (0.4)	0.848
Rheumatoid arthritis	4,775 (7.7)	3,134 (8.0)	1,641 (7.1)	<0.001
Seronegative spondyloarthropathies	2,287 (3.7)	1,370 (3.5)	917 (4.0)	0.005
Systemic lupus erythematosus	460 (0.7)	295 (0.8)	165 (0.7)	0.565
Other systemic connective tissue disorders	412 (0.7)	277 (0.7)	135 (0.6)	0.065
Vasculitis	1,268 (2.0)	812 (2.1)	456 (2.0)	0.345
Other comorbidities				
Asthma	5,673 (9.1)	3,735 (9.6)	1938 (8.4)	<0.001
Chronic obstructive pulmonary disease	22,347 (35.9)	15,253 (39.1)	7,094 (30.6)	<0.001
Diabetes mellitus	29,096 (46.8)	19,165 (49.1)	9,931 (42.9)	<0.001
Hyperlipidemia	54,417 (87.5)	34,135 (87.5)	20,282 (87.5)	1.00
Hypertension	58,873 (94.7)	37,167 (95.3)	21,706 (93.7)	<0.001
Hypothyroidism	9,723 (15.6)	6,199 (15.9)	3,524 (15.2)	0.023
Liver disease, mild	4,635 (7.5)	2,942 (7.5)	1,693 (7.3)	0.283
Liver disease, moderate, or severe	982 (1.6)	688 (1.8)	294 (1.3)	<0.001
Charlson Comorbidity Index, median (IQR)	4 (2-6)	4 (2-6)	3 (2-6)	<0.001
1-2	16,188 (26.0)	9,250 (23.7)	6,938 (29.9)	<0.001
3-5	23,462 (37.7)	14,565 (37.3)	8,897 (38.4)	
>5	22,532 (36.2)	15,191 (38.9)	7,341 (31.7)	
Frailty	13,701 (22.0)	9,094 (23.3)	4,607 (19.9)	<0.001
Smoking status, n (%)				
Current smoker	12,228 (19.7)	8,260 (21.2)	3,968 (17.1)	<0.001
Past smoker	38,273 (61.5)	23,791 (61.0)	14,482 (62.5)	
Never smoker	10,506 (16.9)	6,240 (16.0)	4,266 (18.4)	
Missing	1,175 (1.9)	715 (1.8)	460 (2.0)	
Medication therapy, n (%)				
Angiotensin-converting enzyme inhibitors/angiotensin receptor blockers	40,796 (65.6)	25,560 (65.5)	15,236 (65.7)	0.596
Anticoagulants	11,624 (18.7)	7,958 (20.4)	3,666 (15.8)	<0.001
Antihyperlipidemic	47,055 (75.7)	29,073 (74.5)	17,982 (77.6)	<0.001
Statin				
High intensity	21,440 (34.5)	12,824 (32.9)	8,616 (37.2)	<0.001
Moderate intensity	22,991 (37.0)	14,516 (37.2)	8,475 (36.6)	
Low intensity	1799 (2.9)	1,200 (3.1)	599 (2.6)	
No statin use	15,952 (25.7)	10,466 (26.8)	5,486 (23.7)	
Antiplatelet	38,590 (62.1)	24,212 (62.1)	14,378 (62.0)	0.939
Colchicine	3,118 (5.0)	2082 (5.3)	1,036 (4.5)	<0.001
Glucagon-like peptide-1 receptor agonists (GLP-1RA)	1,321 (2.1)	829 (2.1)	492 (2.1)	0.439
Sodium-glucose transport protein 2 inhibitors (SGLT-2i)	1862 (3.0)	1,165 (3.0)	697 (3.0)	0.903
Systemic corticosteroids	17,264 (27.8)	11,479 (29.4)	5,785 (25.0)	<0.001
Physiologic measures, mean (SD)				
Body mass index, mean (SD)	30.2 (5.8)	30.9 (6.0)	29.1 (5.1)	<0.001
Missing, n (%)	926 (1.5)	551 (1.4)	375 (1.6)	0.044
Systolic blood pressure, mean (SD)	135.2 (13.7)	135.3 (13.7)	135.0 (13.6)	0.018
Missing, n (%)	539 (0.9)	293 (0.8)	246 (1.1)	<0.001
Diastolic blood pressure, mean (SD)	74.1 (8.7)	74.2 (8.8)	74.1 (8.6)	0.072
Missing, n (%)	539 (0.9)	293 (0.8)	246 (1.1)	<0.001
Laboratory values, mean (SD)				
Hemoglobin A1c (%)	13.2 (1.8)	13.2 (1.8)	13.3 (1.8)	<0.001
Missing, n (%)	8,368 (13.5)	5,002 (12.8)	3,366 (14.5)	<0.001
eGFR (based on serum creatinine)	49.3 (12.3)	49.0 (12.6)	49.8 (11.9)	<0.001
Missing, n (%)	316 (0.5)	201 (0.5)	115 (0.5)	0.791
uACR	174.09 (1,569.16)	178.63 (469.67)	167.03 (2,437.98)	0.522
Missing, n (%)	30,704 (49.4)	19,858 (50.9)	10,846 (46.8)	<0.001
LDL cholesterol	87.80 (30.67)	89.03 (31.12)	85.72 (29.80)	<0.001
Missing, n (%)	2,719 (4.4)	1,637 (4.2)	1,082 (4.7)	0.006
Northeast	8,674 (13.9)	5,272 (13.5)	3,402 (14.7)	<0.001
Midwest	7,028 (11.3)	4,476 (11.5)	2,552 (11.0)	
South	26,691 (42.9)	17,010 (43.6)	9,681 (41.8)	
West	18,488 (29.7)	11,454 (29.4)	7,034 (30.4)	
Noncontinental USA^a^	1,301 (2.1)	794 (2.0)	507 (2.2)	

CDC = Centers for Disease Control; eGFR = estimated glomerular filtration rate; hsCRP = high sensitivity c-reactive protein; LDL = low-density lipoprotein; SVI = Social vulnerability index; TIA = transient ischemic stroke; uACR = urine albumin creatinine ratio.

a Alaska, Hawaii, Puerto Rico, U.S. Virgin Islands.

Across Veterans with and without SI, there were similar rates of angiotensin-converting enzyme inhibitor/angiotensin receptor blocker therapy (65.5% [25,560/39,006] vs 65.7% [15,236/23,176]; *P* = 0.60). Veterans with SI were less likely to be on lipid-lowering therapy (74.5% [29,073/39,006] vs 77.6% [17,892/23,176]; *P* < 0.001). Across both groups, glucagon-like peptide-1 receptor agonist treatment (2.1% [829/39,006] vs 2.1% [492/23,176]; *P* = 1.0) and sodium-glucose cotransporter-2 inhibitors treatment (3.0% [1,165/39,006] vs 3.0% [697/23,176]; *P* = 0.90) were rare.

### Clinical outcomes

**Figure 2:**
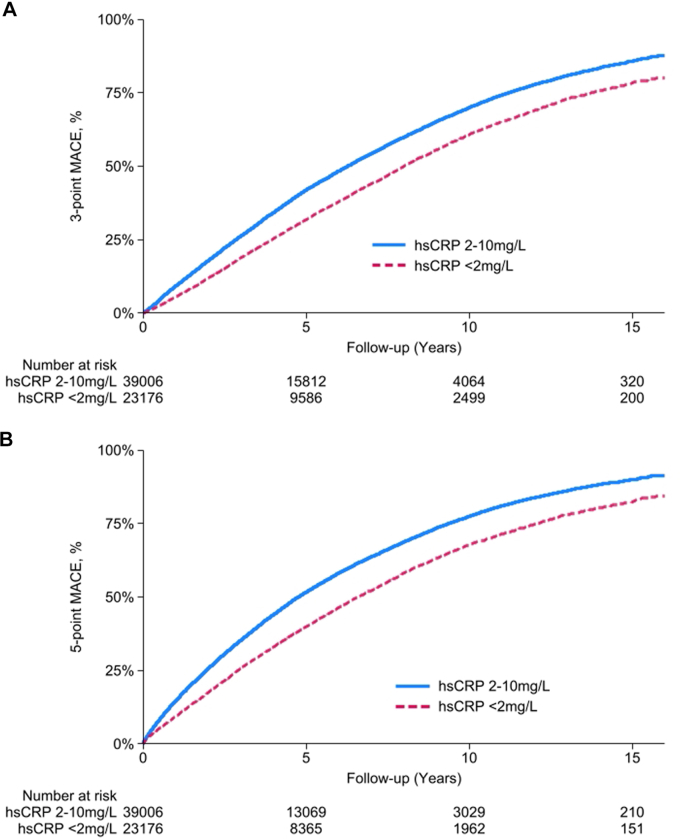
**Association Between Systemic Inflammation and Clinical Outcomes** (A) Displays the cumulative incidence of the 3-point major adverse cardiovascular event outcome (death, myocardial infarction, and stroke) based on hsCRP level. (B) Displays the cumulative incidence of 5-point MACE based on the hsCRP level. MACE = major adverse cardiovascular events; other abbreviation as in Figure 1.

The median follow-up duration was 4.2 years (IQR: 2.1-7.0 years). The total duration of follow-up was 307,074 person-years. The primary 3-point MACE outcome (all-cause death/MI/stroke) rate was 11.64 per 100 patient-years (100PY) (95% CI: 11.48-11.79) among those with SI compared with 8.66 (95% CI: 8.49-8.83) among those without SI (*P* < 0.001) (Table 2). Figure 2 displays the cumulative incidence curves stratified by SI status. After adjustment for age, sex, index year, comorbidities, and medications, the HR between SI and the 3-point MACE outcome was 1.40 (95% CI: 1.36-1.43) (Central Illustration). With hsCRP levels divided into quartiles, there was a stepwise significant association between higher hsCRP and 3-point MACE (Supplemental Table 5). Supplemental Figure 2 displays an approximately linear association between hsCRP modeled as a spline and 3-point MACE.

**Table 2:** Rates of Adverse Cardiovascular Outcomes With and Without SI (With 95% CIs)^a^

Cardiovascular Outcome	Overall (N = 62,182)	With SI (hsCRP ≥2 and ≤10 mg/L) (n = 39,006)	Without SI (hsCRP<2 mg/L) (n = 23,176)	*P* Value^b^	HR with SI	
					Model A^c^	Model B^d^
3-point ASCVD MACE^e^	10.52 (10.40-10.63)	11.64 (11.48-11.79)	8.66 (8.49-8.83)	<0.001	1.48 (1.44-1.51)^a^	1.40 (1.36-1.43)^a^
5-point ASCVD MACE^f^	13.65 (13.52-13.79)	15.28 (15.09-15.46)	11.07 (10.87-11.27)	<0.001	1.47 (1.44-1.50)^a^	1.41 (1.38-1.44)^a^
All-cause death	10.07 (9.95-10.17)	10.52 (10.37-10.66)	9.31 (9.14-9.48)	<0.001	1.27 (1.24-1.30)^a^	1.19 (1.16-1.22)^a^
Cardiovascular death	3.92 (3.84-3.99)	4.16 (4.07-4.25)	3.50 (3.39-3.61)	<0.001	1.32 (1.26-1.37)^a^	1.22 (1.18-1.27)^a^
Ischemic stroke or TIA	0.72 (0.69-0.75)	0.78 (0.75-0.82)	0.61 (0.57-0.66)	<0.001	1.33 (1.21-1.45)^a^	1.30 (1.19-1.43)^a^
Myocardial infarction	0.91 (0.88-0.94)	0.97 (0.93-1.01)	0.81 (0.76-0.86)	<0.001	1.22 (1.12-1.32)^a^	1.18 (1.09-1.28)^a^
Coronary revascularization	2.78 (2.71-2.83)	3.07 (2.99-3.15)	2.29 (2.2-2.37)	<0.001	1.10 (1.05-1.15)^a^	1.09 (1.04-1.14)^a^
Peripheral arterial revascularization	2.48 (2.42-2.53)	2.73 (2.65-2.81)	2.07 (1.98-2.15)	<0.001	1.28 (1.22-1.34)^a^	1.24 (1.18-1.31)^a^

ASCVD = atherosclerotic cardiovascular disease; MACE = major adverse cardiovascular outcomes; SI = systemic inflammation; other abbreviations as in Table 1.

a Indicates *P* < 0.05 for HRs evaluating association between SI (hsCRP≥2 mg/L and ≤10 mg/L) and the outcome.

b The *P* value for the difference in incidence rates between Veterans with SI and without SI.

c Model A adjusted for age, sex, and year of hsCRP measurement.

d Model B adjusted for age, sex, year of hsCRP measurement, Charlson Comorbidity Index, prior MI, prior coronary revascularization, smoking status, body mass index, eGFR, lipid-lowering therapies, antiplatelets, anticoagulants, angiotensin-converting enzyme inhibitors/angiotensin receptor blockers, SGLT2i, and GLP-1 RA.

e 3-point MACE includes all-cause death, MI, and ischemic stroke or TIA.

f 5-point MACE includes all-cause death, MI, ischemic stroke or TIA, coronary revascularization, and peripheral artery revascularization.

**Figure 3:**
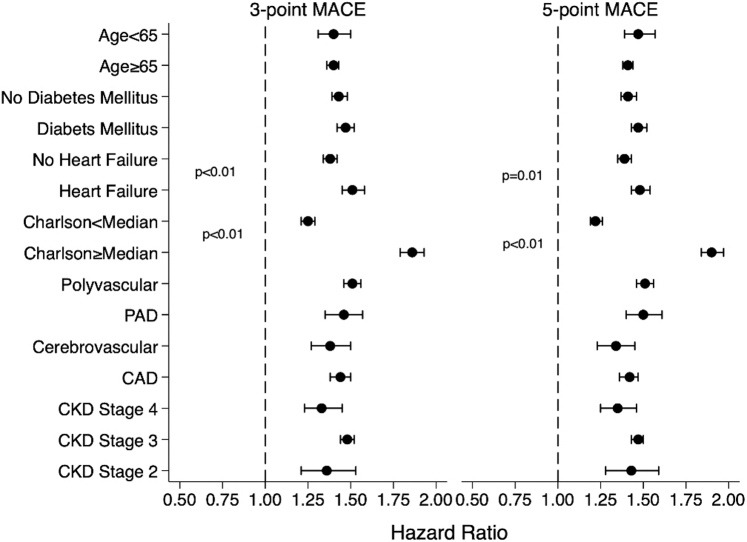
**Adjusted Association Between Systemic Inflammation and Major Adverse Cardiovascular Events Stratified by Veteran Characteristics∗** ∗The *P* values for significant interactions (*P* < 0.05) between subgroup characteristic and the SI exposure are displayed. All other interaction terms were not significant. CAD = coronary artery disease; PAD = peripheral arterial disease; other abbreviations as in Figures 1 and 2.

**Central Illustration:**
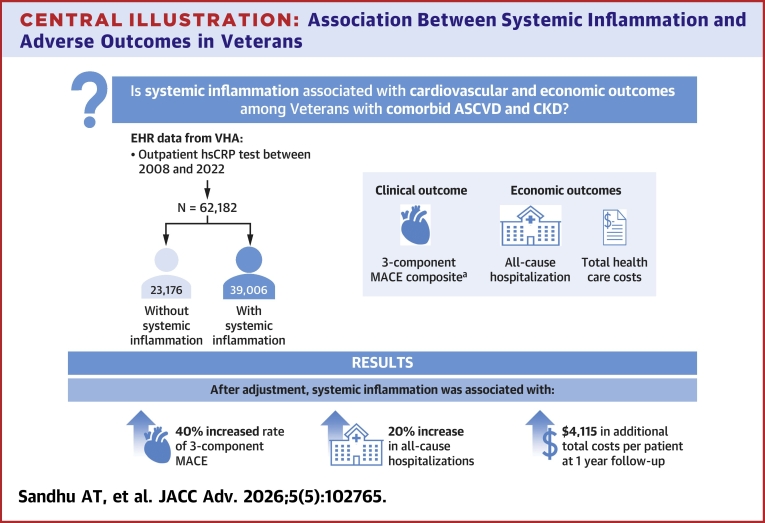
**Association Between Systemic Inflammation and Adverse Outcomes in Veterans** ^a^All-cause death, MI, and stroke. ASCVD = atherosclerotic cardiovascular disease; CKD = chronic kidney disease; EHR = electronic health record; hsCRP = high-sensitivity C-reactive protein; MACE = major adverse cardiovascular events; VHA = Veterans Health Administration.

The 5-point composite MACE outcome (all-cause death/MI/stroke/coronary revascularization/peripheral arterial revascularization) rate was 15.28 per 100PY (95% CI: 15.09-15.46) among those with SI compared with 11.07 (95% CI: 10.87-11.27) among those without SI (*P* < 0.001) (Table 2). After adjustment, SI was associated with an increased hazard of the 5-point MACE outcome (HR: 1.41; 95% CI: 1.38-1.44). SI was also significantly associated with each individual component of the composite outcome (Table 2).

The cardiovascular death rate was 4.16 per 100PY (95% CI: 4.07-4.25) among those with SI compared with 3.50 (95% CI: 3.39-3.61) among those without SI (*P* < 0.001). After adjustment, SI was associated with an increased hazard of cardiovascular death (HR: 1.22; 95% CI: 1.18-1.27) (Table 2).

We tested the interaction between key clinical characteristics and SI on the 3-point MACE outcome. We found the adjusted association between SI and 3-point MACE was consistent across age, diabetes mellitus, ASCVD type, and CKD stage (Figure 3). We found the association between SI and the 3-point MACE outcome was stronger among those with heart failure (HR: 1.41; 95% CI: 1.35-1.46) compared with those without heart failure (HR: 1.26; 95% CI: 1.22-1.30) (*P* < 0.001). We also found the association between SI and the 3-point MACE outcome was stronger among those with CCI score above the median of 4 (HR: 1.73; 95% CI: 1.67-1.79) compared with those with CCI score below the median (HR: 1.14; 95% CI: 1.10-1.18) (*P* < 0.001). The results were overall similar for the 5-point MACE outcome (Figure 3).

We performed multiple sensitivity analyses. First, the results were similar in a complete case analysis that excluded individuals with missing eGFR or body mass index data (Supplemental Table 6). In another sensitivity analysis, after excluding the 8,592 Veterans with systemic autoimmune conditions from the study cohort, we found the 3-point MACE rate remained significantly higher among the 33,503 Veterans with SI (12.09 per 100PY; 95% CI: 11.94-12.25) compared with the 20,087 Veterans without SI (10.09 per 100PY; 95% CI: 9.88-10.29) (*P* < 0.001). The results were similar for each of the other clinical endpoints (Supplemental Table 7). Second, after adjustment for all covariates with baseline imbalance, we found a similar association between SI and 3-point MACE (HR: 1.36; 95% CI: 1.33-1.39). Finally, after including Veterans with hsCRP >10 mg/L, cardiovascular event rates increased among Veterans with hsCRP >2 mg/L (Supplemental Table 8).

### Acute care utilization and health care cost

We found higher acute care utilization among Veterans with SI (Supplemental Table 9). Among Veterans with 1 year of follow-up, SI was associated with a 24% increase in the rate of all-cause hospitalizations after adjustment for demographics, year of hsCRP, comorbidities, and medications (IRR: 1.24; 95% CI: 1.20-1.29). SI was associated with a 31% increase in the rate of cardiovascular hospitalizations (IRR: 1.31; 95% CI: 1.23-1.39). The associations remained significant across shorter and longer follow-up durations (Table 3).

**Table 3:** Association Between SI and Acute Care Utilization Rate^a^

	6-Month Follow-Up (n = 60,276)^b^		1-Year Follow-Up (n = 57,975)^b^		3-Year Follow-Up (n = 40,524)^b^		5-Year Follow-Up (n = 26,985)^b^	
	Model A^c^	Model B^d^	Model A^c^	Model B^d^	Model A^c^	Model B^d^	Model A^c^	Model B^d^
All-cause hospitalization	1.38 (1.32-1.44)^a^	1.28 (1.23-1.34)^a^	1.34 (1.29-1.39)^a^	1.24 (1.20-1.29)^a^	1.24 (1.20-1.28)^a^	1.15 (1.11-1.18)^a^	1.24 (1.20-1.29)^a^	1.15 (1.11-1.19)^a^
Cardiovascular hospitalization	1.53 (1.42-1.65)^a^	1.41 (1.30-1.52)^a^	1.42 (1.34-1.51)^a^	1.31 (1.23-1.39)^a^	1.25 (1.18-1.31)^a^	1.13 (1.07-1.19)^a^	1.26 (1.20-1.33)^a^	1.17 (1.10-1.23)^a^
Emergency department	1.16 (1.12-1.19)^a^	1.11 (1.08-1.14)^a^	1.14 (1.11-1.16)^a^	1.09 (1.06-1.12)^a^	1.10 (1.07-1.12)^a^	1.04 (1.02-1.07)^a^	1.11 (1.08-1.15)^a^	1.07 (1.04-1.10)^a^

Abbreviation as in Table 2.

a Indicates *P* < 0.05 for incidence rate ratio between SI (hsCRP≥2 mg/L and ≤10 mg/L) and the outcome.

b Each analysis excludes individuals with death or loss to follow-up before the end of the follow-up period.

c Model A adjusted for age, sex, and year of hsCRP measurement.

d Model B adjusted for age, sex, year of hsCRP measurement, Charlson Comorbidity Index, prior MI, prior coronary revascularization, smoking status, body mass index, eGFR, lipid-lowering therapies, antiplatelets, anticoagulants, angiotensin-converting enzyme inhibitors/angiotensin receptor blockers, SGLT2i, and GLP-1 RA.

The median cost per PY was $33,704 (IQR: $15,726 to $69,103) among those with SI and $28,013 (IQR: $13,055 to $58,069) among those without SI (*P* < 0.001). Inpatient, outpatient, pharmacy, and cardiovascular-related costs were all higher among those with SI (Table 4).

**Table 4:** Cost of Care per Patient Year Stratified by SI (2023 USD)

Per Patient-Year of Follow-Up	Overall (N = 62,182) Median (IQR)	Without SI (hsCRP <2) (n = 23,176) Median (IQR)	With SI (hsCRP ≥2 and ≤ 10) (n = 39,006) Median (IQR)	*P* Value^a^
Total	31,459 (14,645-64,963)	28,013 (13,055-58,069)	33,704 (15,726-69,103)	<0.001
Inpatient	8,912 (213-30,103)	6,899 (0-24,871)	10,219 (671-33,317)	<0.001
Cardiovascular	3,502 (763-12,924)	2,822 (644-10,858)	3,994 (845-14,289)	<0.001
Outpatient	14,778 (7,770-26,943)	13,791 (7,232-25,278)	15,369 (8,120-27,796)	<0.001
Pharmacy	1,571 (654-3,467)	1,432 (572-3,288)	1,658 (709-3,554)	<0.001

USD = U.S. dollars; other abbreviations as in Tables 1 and 2.

a The *P* value for the difference in costs per patient-year between Veterans with SI and without SI.

Over 1 year of follow-up, SI was associated with $4,115 (95% CI: $1,878-$6,353) in additional total costs and $2,179 (95% CI: $1,392-$2,966) in inpatient costs after adjustment. For cardiovascular-related costs, SI was associated with $1,346 (95% CI: $779-$1,914) in additional costs. Costs were also significantly greater for outpatient costs and across varying follow-up periods, but were not consistently higher for pharmacy-related costs (Table 5).

**Table 5:** Marginal Cost of SI Based on Follow-Up Duration and Cost Category (USD 2023)^a^^,^^b^

	6-Month Follow-Up		1-Year Follow-Up		3-Year Follow-Up		5-Year Follow-Up	
	Model A^c^	Model B^d^	Model A^c^	Model B^d^	Model A^c^	Model B^d^	Model A^c^	Model B^d^
Total	3,805 (2,477-5,133)^a^	2,759 (1,542-3,976)^a^	6,028 (3,792-8,263)^a^	4,115 (1,878-6,353)^a^	13,382 (10,774-15,991)^a^	7,461 (4,633-10,288)^a^	20,020 (15,718-24,323)^a^	10,831 (6,376-15,287)^a^
Inpatient	2,322 (1,807-2,836)^a^	1,677 (1,154-2,201)^a^	3,430 (2,657-4,203)^a^	2,179 (1,392-2,966)^a^	8,091 (6,356-9,826)^a^	4,793 (3,015-6,572)^a^	12,449 (9,861-15,037)^a^	8,118 (5,526-10,710)^a^
Cardiovascular	1,471 (1,138-1,804)^a^	1,181 (836.5-1,526)^a^	2,095 (1,586-2,604)^a^	1,346 (779-1,914)^a^	4,284 (3,200-5,369)^a^	2,733 (1,568-3,898)^a^	5,888 (4,435-7,342)^a^	4,029 (2,528-5,531)^a^
Outpatient	856 (600-1,112)^a^	470 (200-740)^a^	1,252 (829-1,674)^a^	465 (25-905)^a^	4,092 (2,968-5,217)^a^	1,856 (756-2,956)^a^	5,759 (3,597-7,921)^a^	1,761 (−248.4 to 3,770)
Pharmacy	284 (−161 to 730)	154 (−190 to 498)	716 (−199 to 1,631)	492 (−307 to 1,291)	1,205 (308-2,101)^a^	571 (−362 to 1,503)	1,740 (338-3,141)^a^	810 (−689 to 2,304)

Abbreviations as in Tables 2 and 4.

a Indicates *P* < 0.05 for adjusted marginal cost difference between Veterans with SI (hsCRP≥2 mg/L and ≤10 mg/L) and without SI (hsCRP<2 mg/L).

b Each analysis excludes individuals with death or loss to follow-up before the end of the follow-up period.

c Model A adjusted for age, sex, and year of hsCRP measurement.

d Model B adjusted for age, sex, year of hsCRP measurement, Charlson Comorbidity Index, prior MI, prior coronary revascularization, smoking status, body mass index, eGFR, lipid-lowering therapies, antiplatelets, anticoagulants, angiotensin-converting enzyme inhibitors/angiotensin receptor blockers, SGLT2i, and GLP-1 RA.

## Discussion

We evaluated the association between SI and clinical and economic outcomes among Veterans with comorbid ASCVD and CKD. We found SI was consistently associated with an increased risk of MACE after adjustment for clinical characteristics. Furthermore, SI was associated with more frequent acute care utilization and higher costs. These results demonstrate the substantial clinical and economic burden associated with SI among individuals with ASCVD and CKD.

The importance of the interplay between ASCVD and CKD is increasingly recognized. The American Heart Association (AHA) released a presidential advisory stressing the importance of addressing cardiovascular-kidney-metabolic syndrome, which is a systemic disorder characterized by pathophysiological interactions among metabolic risk factors, CKD, and the cardiovascular system.^3^ Multiple prior analyses have demonstrated the high morbidity of comorbid ASCVD and CKD.^28^, ^29^, ^30^, ^31^, ^32^ In our cohort of Veterans with comorbid ASCVD and CKD, we found an annual mortality rate exceeding 10%, an annual hospitalization rate of 0.76, and median annual costs exceeding $31,000. This clinical and economic burden far exceeds that of the average Medicare patient, reinforcing the burden of comorbid ASCVD and CKD.^33^^,^^34^

Overall Veterans with SI had relatively similar age and median CCI compared with Veterans without SI. After adjustment for clinical characteristics, SI remained significantly associated with an increased risk of MACE. This aligns with prior longitudinal epidemiologic cohorts that demonstrate inflammation, measured by hsCRP, is associated with an increased risk cardiovascular events.^14^^,^^35^ Our analysis provides supportive real-world data that hsCRP measurements during routine clinical practice are also predictive of cardiovascular events. This is consistent with the results of a recent analysis of hsCRP measurements among individuals in Sweden with ASCVD.^36^ Mazhar et al found hsCRP ≥2 mg/L was associated with a significant 30% increase in the hazard of death among individuals with ASCVD. In their cohort, 79% of individuals had eGFR ≥60 mL/min/1.73 m^2^. Our analysis demonstrates that SI remains a strong predictor of outcomes in a higher-risk cohort with the comorbid risk of CKD. Furthermore, we demonstrate that the increase in adverse clinical outcomes also translates to a substantial increase in economic burden.

Statin therapy is the cornerstone of secondary ASCVD prevention, and statins reduce hsCRP levels in addition to low-density lipoprotein cholesterol.^37^ Across 3 separate clinical trials of patients on statin therapy, residual SI risk—based on hsCRP—was a stronger predictor of adverse cardiovascular events than residual cholesterol risk.^38^ This has also been demonstrated among patients on nonstatin lipid-lowering therapy.^10^ Overall the cohort in the present study was well treated with lipid-lowering therapy; 74% were on statin therapy, and the mean low-density lipoprotein level was 87 mg/dL. Despite the high statin use, we found a high prevalence of SI, which was consistently associated with adverse clinical and economic outcomes.

Our data support hsCRP screening as part of secondary ASCVD prevention to better identify those at the highest risk of recurrent events. Although the 2018 American College of Cardiology/AHA Cholesterol Treatment Guidelines recognize elevated hsCRP as a risk enhancer that can guide decisions to intensify statin therapy,^39^ the 2023 American College of Cardiology/AHA Guideline for the Management of Chronic Coronary Disease do not have a recommendation for hsCRP testing to guide risk stratification for secondary prevention.^40^ In contrast, the 2024 European Society of Cardiology Guidelines for Chronic Coronary Syndrome include a IIA recommendation to consider hsCRP testing to improve risk stratification and guide treatment.^41^ Furthermore, the European Association of Preventive Cardiology recommend estimating risk of recurrent ASCVD events using the SMART2 risk score, which incorporates hsCRP levels in risk prediction.^42^^,^^43^ Identifying those at an elevated risk of recurrent ASCVD has multiple potential benefits. First, an individual’s risk of recurrent ASCVD should be incorporated into patient counseling regarding potential treatments and lifestyle.^41^ Second, experts have suggested more aggressive low-density lipoprotein cholesterol reduction among patients with ASCVD at a very high risk for recurrent events.^44^

Multiple trials have evaluated therapies to specifically target SI among individuals with ASCVD. Canakinumab, an antibody targeting interleukin-1β, reduced ASCVD events,^45^ but it did not receive an Food and Drug Administration indication for secondary ASCVD prevention. The results of trials evaluating colchicine for secondary ASCVD prevention have been mixed. 2 large outcome trials demonstrated the benefit of colchicine among patients with CAD,^46^^,^^47^ colchicine did not improve outcomes among patients in the largest trial to date.^48^ One hypothesis posited for the negative results of the CLEAR SYNERGY trial is the magnitude of hsCRP reduction was insufficient given the mean hsCRP remained above 2 mg/L at 3 months in the colchicine arm.^49^ Several ongoing trials are investigating the potential of targeting IL-6 with ziltivekimab, a monoclonal antibody against IL-6 that reduces hsCRP levels by 88%.^50^ This includes the ongoing ZEUS trial, which is testing the efficacy of ziltivekimab among individuals with comorbid ASCVD and CKD.

### Study Limitations

There are multiple limitations to this study. First, as an observational study, there may be additional unmeasured variables that may have confounded the association between hsCRP levels and cardiovascular outcomes. Noncardiovascular comorbidities that influence SI may also contribute directly to increased adverse outcomes and increased health care costs. However, we saw similar associations between hsCRP and cardiovascular-specific outcomes. Second, as a real-world cohort, the clinical decision to measure hsCRP may reflect additional clinical concern that may increase the absolute risk of the cohort, although hsCRP was measured in both comparison arms. We have described differences between our study cohort and the overall VA population without hsCRP testing that influence the generalizability of our findings. Third, the use of nonadjudicated outcomes may introduce misclassification of events; notably, each of the classification approaches used in this study has been validated previously. Fourth, although the distribution of sex was similar between arms, the overall proportion of women was low. In addition, Veterans cohorts have high burden of cardiovascular disease and noncardiovascular comorbidities. These 2 factors influence the generalizability of this study to broader cohorts and emphasize the value of validating these findings in different populations. Finally, we used a broad study time period to maximize the sample size and duration of follow-up. In this context, therapy rates with glucagon-like peptide-1 receptor agonists and sodium-glucose transport protein 2 inhibitors are lower than currently observed, which is important given the known cardiovascular and renal benefits of both therapy classes.

## Conclusions

Our analysis highlights the significant association between SI, adverse cardiovascular events, and increased health care costs among Veterans with comorbid ASCVD and CKD. These findings suggest the importance of recognizing SI as a marker of increased risk among this high-risk patient population. Ongoing studies will elucidate potential targeted interventions to lower this substantial clinical risk and economic burden.

Perspectives **COMPETENCY IN MEDICAL KNOWLEDGE:** This study finds that the presence of elevated hsCRP, a marker of SI, is associated with increased rates of adverse cardiovascular outcomes and increased health care utilization among individuals with comorbid ASCVD and CKD. This supports the measurement of elevated hsCRP to identify patients at an increased risk of recurrent cardiovascular events.

**TRANSLATIONAL OUTLOOK:** Future prospective studies should evaluate the potential impact of screening patients with cardiovascular disease and CKD for elevated hsCRP levels. Furthermore, ongoing studies are testing the impact of anti-inflammatory medications on outcomes among patients with SI and comorbid ASCVD and CKD.

## Funding support and author disclosures

This work was funded by 10.13039/501100004191Novo Nordisk, Inc. This material is the result of work supported with resources and the use of facilities at the VA Palo Alto Healthcare System, Palo Alto, CA. Support for the Department of Veterans Affairs (VA) and Centers for Medicare and Medicaid Services data were provided by the VA and the VA Information Resource Center. The funders contributed to the design of the study, interpretation of the data, and review of the manuscript. The funders had no role in the collection, management, analysis, or the decision to submit the manuscript for publication. Dr Sandhu reports research funding outside the submitted work from NIH, the American Heart Association, Amgen, Astra Zeneca, Bayer, and Novartis; and consulting fees from Cleerly, Holosis Health, and Reprieve Cardiovascular. Dr Rodriguez reports equity from Carta Healthcare and HealthPals; and consulting fees from HealthPals, Novartis, NovoNordisk, Esperion Therapeutics, Movano Health, Kento Health, Inclusive Health, Edwards, Arrowhead Pharmaceuticals, Cleerly, iRhythm, and HeartFlow outside the submitted work. Dr Maron reports equity from Ablative Solutions, consultant fees from Regeneron and Scilex, and research funding outside of the submitted work from Cleerly and Omada Health. Dr Zitko and Dr Tonnu-Mihara were employees of Novo Nordisk during the study. Dr Khachatourian is an employee and stockholder of Novo Nordisk, Inc. Dr Skaar is an employee and stockholder of Novo Nordisk, Inc. and holds equity in Trinity Life Sciences. All other authors have reported that they have no relationships relevant to the contents of this paper to disclose.

## References

[1] Centers for Disease Control (2023. Accessed February 1, 2026). Chronic kidney disease in the United States. https://www.cdc.gov/kidney-disease/php/data-research/index.html.

[2] Alanaeme C.J., Bittner V., Brown T.M. (2022). Estimated number and percentage of US adults with atherosclerotic cardiovascular disease recommended add-on lipid-lowering therapy by the 2018 AHA/ACC multi-society cholesterol guideline. Am Heart J Plus.

[3] Ndumele C.E., Rangaswami J., Chow S.L. (2023). Cardiovascular-kidney-metabolic health: a presidential advisory from the American Heart Association. Circulation.

[4] Cherney D.Z.I., Repetto E., Wheeler D.C. (2020). Impact of cardio-renal-metabolic comorbidities on cardiovascular outcomes and mortality in type 2 diabetes mellitus. Am J Nephrol.

[5] Chou C.-L., Chiu H.-W., Hsu Y.-H., Yu S.M.-W., Liou T.-H., Sung L.-C. (2024). Impact of chronic kidney disease and end-stage renal disease on the mid-term adverse outcomes in diabetic patients with cardiovascular diseases. Sci Rep.

[6] Lv L., Rajpura J., Liu M. (2025). Prevalence and clinical characteristics of patients with hsCRP testing and test-confirmed systemic inflammation among individuals with atherosclerotic cardiovascular disease with or without chronic kidney disease in the United States (PLUTUS). Am J Prev Cardiol.

[7] Kaptoge S., Di Angelantonio E., Lowe G. (2010). C-reactive protein concentration and risk of coronary heart disease, stroke, and mortality: an individual participant meta-analysis. Lancet (London, England).

[8] Zhuang Q., Shen C., Chen Y. (2019). Association of high sensitive C-reactive protein with coronary heart disease: a Mendelian randomization study. BMC Med Genet.

[9] Wang A., Liu J., Li C. (2017). Cumulative exposure to high-sensitivity C-Reactive protein predicts the risk of cardiovascular disease. J Am Heart Assoc.

[10] Ridker P.M., Lei L., Louie M.J. (2024). CLEAR outcomes investigators. Inflammation and cholesterol as predictors of cardiovascular events among 13 970 contemporary high-risk patients with statin intolerance. Circulation.

[11] Levey A.S., Stevens L.A., Schmid C.H. (2009). A new equation to estimate glomerular filtration rate. Ann Intern Med.

[12] Barzilay J.I., Farag Y.M.K., Durthaler J. (2024). Albuminuria: an underappreciated risk factor for cardiovascular disease. J Am Heart Assoc.

[13] Ridker P.M. (2016). A test in context: high-sensitivity C-Reactive protein. J Am Coll Cardiol.

[14] Ridker P.M., Hennekens C.H., Buring J.E., Rifai N. (2000). C-Reactive protein and other markers of inflammation in the prediction of cardiovascular disease in women. N Engl J Med.

[15] Maluf C.B., Barreto S.M., Giatti L. (2020). Association between C reactive protein and all-cause mortality in the ELSA-Brasil cohort. J Epidemiol Community Health.

[16] Parrinello C.M., Lutsey P.L., Ballantyne C.M., Folsom A.R., Pankow J.S., Selvin E. (2015). Six-year change in high-sensitivity C-reactive protein and risk of diabetes, cardiovascular disease, and mortality. Am Heart J.

[17] Centers for Disease Control (2023. Accessed February 1, 2026). CDC/ATSDR Social Vulnerability Index (SVI). https://www.atsdr.cdc.gov/place-health/php/svi/index.html.

[18] Kohsaka S., Sandhu A.T., Parizo J.T., Shoji S., Kumamamru H., Heidenreich P.A. (2020). Association of diagnostic coding-based frailty and outcomes in patients with heart failure: a report from the veterans affairs health system. J Am Heart Assoc.

[19] Kokotailo R.A., Hill M.D. (2005). Coding of stroke and stroke risk factors using International classification of diseases, revisions 9 and 10. Stroke.

[20] Metcalfe A., Neudam A., Forde S. (2013). Case definitions for acute myocardial infarction in administrative databases and their impact on In-Hospital mortality rates. Health Serv Res.

[21] Olubowale O.T., Safford M.M., Brown T.M. (2017). Comparison of expert adjudicated coronary heart disease and cardiovascular disease mortality with the National death index: results from the REasons for geographic and racial differences in stroke (REGARDS) study. J Am Heart Assoc.

[22] Lloyd-Jones D.M., Martin D.O., Larson M.G., Levy D. (1998). Accuracy of death certificates for coding coronary heart disease as the cause of death. Ann Intern Med.

[23] Wagner T.H., Chen S., Barnett P.G. (2003). Using average cost methods to estimate encounter-level costs for medical-surgical stays in the VA. Med Care Res Rev.

[24] Centers for Medicare & Medicaid Services NHE deflator - intermediate summary. https://www.cms.gov/research-statistics-data-and-systems/statistics-trends-and-reports/nationalhealthexpenddata/downloads/nhe-deflator.pdf.

[25] Stensrud M.J., Hernán M.A. (2020). Why test for proportional hazards?. JAMA.

[26] Austin P.C., Fine J.P. (2017). Practical recommendations for reporting fine-gray model analyses for competing risk data. Stat Med.

[27] Xu G., Song M., Zhou X., Wu Y., Pazaris M., Spiegelman D. (Posted online October 30, 2021). The missing covariate indicator method is nearly valid almost always. Preprint.

[28] Rashidi A., Sehgal A.R., Rahman M., O’Connor A.S. (2008). The case for chronic kidney disease, diabetes mellitus, and myocardial infarction being equivalent risk factors for cardiovascular mortality in patients older than 65 years. Am J Cardiol.

[29] Wattanakit K., Coresh J., Muntner P., Marsh J., Folsom A.R. (2006). Cardiovascular risk among adults with chronic kidney disease, with or without prior myocardial infarction. J Am Coll Cardiol.

[30] Manjunath G., Tighiouart H., Ibrahim H. (2003). Level of kidney function as a risk factor for atherosclerotic cardiovascular outcomes in the community. J Am Coll Cardiol.

[31] Weiner D.E., Tighiouart H., Stark P.C. (2004). Kidney disease as a risk factor for recurrent cardiovascular disease and mortality. Am J Kidney Dis.

[32] Anavekar N.S., McMurray J.J.V., Velazquez E.J. (2004). Relation between renal dysfunction and cardiovascular outcomes after myocardial infarction. N Engl J Med.

[33] Reschovsky J.D., Hadley J., Saiontz-Martinez C.B., Boukus E.R. (2011). Following the money: factors associated with the cost of treating high-cost medicare beneficiaries. Health Serv Res.

[34] Krumholz H.M., Nuti S.V., Downing N.S., Normand S.-L.T., Wang Y. (2015). Mortality, hospitalizations, and expenditures for the medicare population age 65 and older, 1999–2013. JAMA.

[35] Ridker P.M., Cushman M., Stampfer M.J., Tracy R.P., Hennekens C.H. (1997). Inflammation, aspirin, and the risk of cardiovascular disease in apparently healthy men. N Engl J Med.

[36] Mazhar F., Faucon A.-L., Fu E.L. (2024). Systemic inflammation and health outcomes in patients receiving treatment for atherosclerotic cardiovascular disease. Eur Heart J.

[37] Ridker P.M., Cannon C.P., Morrow D. (2005). Pravastatin or atorvastatin evaluation and infection therapy-thrombolysis in myocardial infarction 22 (PROVE IT-TIMI 22) investigators. C-reactive protein levels and outcomes after statin therapy. N Engl J Med.

[38] Ridker P.M., Bhatt D.L., Pradhan A.D., Glynn R.J., MacFadyen J.G., Nissen S.E. (2023). Inflammation and cholesterol as predictors of cardiovascular events among patients receiving statin therapy: a collaborative analysis of three randomised trials. Lancet.

[39] Grundy S.M., Stone N.J., Bailey A.L. (2019). 2018 AHA/ACC/AACVPR/AAPA/ABC/ACPM/ADA/AGS/APhA/ASPC/NLA/PCNA guideline on the management of blood cholesterol: executive summary: a report of the American College of Cardiology/American Heart Association task force on clinical practice guidelines. J Am Coll Cardiol.

[40] Virani S.S., Newby L.K., Arnold S.V. (2023). 2023 AHA/ACC/ACCP/ASPC/NLA/PCNA guideline for the management of patients with chronic coronary disease: a report of the American Heart Association/American College of Cardiology Joint Committee on clinical practice guidelines. Circulation.

[41] Vrints C., Andreotti F., Koskinas K.C. (2024). 2024 ESC guidelines for the management of chronic coronary syndromes. Eur Heart J.

[42] Rossello X., Dorresteijn J.A., Janssen A. (2019). Risk prediction tools in cardiovascular disease prevention: a report from the ESC prevention of CVD programme led by the European Association of Preventive Cardiology (EAPC) in collaboration with the Acute Cardiovascular Care Association (ACCA) and the Association of Cardiovascular Nursing and Allied Professions (ACNAP). Eur J Prev Cardiol.

[43] Hageman S.H.J., McKay A.J., Ueda P. (2022). Estimation of recurrent atherosclerotic cardiovascular event risk in patients with established cardiovascular disease: the updated SMART2 algorithm. European Heart Journal.

[44] Lloyd-Jones D.M., Morris P.B., Ballantyne C.M. (2022). 2022 ACC expert consensus decision pathway on the role of nonstatin therapies for LDL-cholesterol lowering in the management of atherosclerotic cardiovascular disease risk: a report of the American College of Cardiology solution set oversight committee. J Am Coll Cardiol.

[45] Ridker P.M., Everett B.M., Thuren T. (2017). Antiinflammatory therapy with canakinumab for atherosclerotic disease. N Engl J Med.

[46] Tardif J.-C., Kouz S., Waters D.D. (2019). Efficacy and safety of low-dose colchicine after myocardial infarction. N Engl J Med.

[47] Nidorf S.M., Fiolet A.T.L., Mosterd A. (2020). Colchicine in patients with chronic coronary disease. N Engl J Med.

[48] Jolly S.S., d’Entremont M.-A., Lee S.F. (2025). Colchicine in acute myocardial infarction. N Engl J Med.

[49] Misra A., Psaltis P.J., Mondal A.R., Nelson A.J., Nidorf S.M. (2025). Implications and limitations of the CLEAR-SYNERGY trial for the use of low-dose colchicine in cardiovascular disease. Nat Cardiovasc Res.

[50] Ridker P.M., Devalaraja M., Baeres F.M.M. (2021). IL-6 inhibition with ziltivekimab in patients at high atherosclerotic risk (RESCUE): a double-blind, randomised, placebo-controlled, phase 2 trial. Lancet (London, England).

