# Supplementary material for: Systemic Inflammation and Adverse Outcomes in Patients With Atherosclerotic Cardiovascular Disease and Chronic Kidney Disease
Source: JACC Adv. 2026 Apr 22;5(5):102765. doi: 10.1016/j.jacadv.2026.102765 (PMC13126473; doi:10.1016/j.jacadv.2026.102765)
Supplement: Supplemental Material [file mmc1.docx]

**SUPPLEMENT**

**Table of Contents**

Table of Contents

[Supplemental Methods. 3](#_Toc223455911)

[Supplement Table 1. Code List for Comorbidities, Laboratory Tests, and Procedures 4](#_Toc223455912)

[Supplement Table 2. Comparison of Patient Characteristics Excluded Due to Comorbidities 12](#_Toc223455913)

[Supplement Table 3. Comparison of Patient Characteristics with and Without hsCRP Testing 14](#_Toc223455914)

[Supplement Table 4. Comparison of Veterans with hsCRP>10 and hsCRP >2 and <10mg/L 16](#_Toc223455915)

[Supplemental Table 5. Association Between hsCRP Quartiles and Adverse Cardiovascular Outcomes 20](#_Toc223455916)

[Supplement Table 6. Rates of Adverse Cardiovascular Outcomes with and without SI with Complete Case Analysis(with 95% Confidence Intervals)*^, †^ 22](#_Toc223455917)

[Supplemental Table 7. Rates of Adverse Cardiovascular Outcomes among Veterans with and without SI Excluding Individuals with Systemic Autoimmune Conditions 23](#_Toc223455918)

[Supplemental Table 8. Rates of Adverse Cardiovascular Outcomes with and without SI Including hsCRP>10mg/L 24](#_Toc223455919)

[Supplement Table 9. Annual Rates of Acute Care Utilization among Veterans with and without SI 25](#_Toc223455920)

[Supplement Figure 1. Histogram of hsCRP Levels 26](#_Toc223455921)

[Supplement Figure 2. Association Between hsCRP Modeled as a Spline and 3-point MACE 27](#_Toc223455922)

# **Supplemental Methods.**

*Imputation of Data.* The following clinical characteristics were included in adjusted models: age, sex, year of hsCRP measurement, Charlson comorbidity index, prior MI, prior coronary revascularization, smoking status, body mass index, eGFR, and the following medications: lipid-lowering therapies, antiplatelets, anticoagulants, angiotensin-converting enzyme inhibitors/angiotensin receptor blockers, SGLT2 inhibitors, and GLP-1 receptor agonists.

All variables were non-missing except for BMI and eGFR. Across these variables we identified the following missingness pattern:

| Frequency | GFR | BMI |
| --- | --- | --- |
| 98% (60,961/62,182) | Non-Missing | Non-Missing |
| 1.5% (905/62,182) | Non-Missing | Missing |
| 0.5% (295 /62,182) | Missing | Non-Missing |
| <0.1% (21/62,182) | Missing | Missing |

Predicting missing BMI with a logistic regression model with other available characteristics yielded a pseudo-R^2^ of 0.14. Predicting missing GFR with other available characteristics yielded a pseudo-R^2^ of 0.15.

Given the low rates of missingness, we performed mean imputation. We also included missing indicators given the data is likely not missing completely at random to account for potential non-random missingness. Furthermore, we performed a complete case sensitivity analysis.

**Supplement Table 1. Code List for Comorbidities, Laboratory Tests, and Procedures**

| **Condition** | **ICD Code List** |
| --- | --- |
| Diabetes Mellitus | 24900; 24901; 24910; 24911; 24920; 24921; 24930; 24931; 24940; 24941; 24950; 24951; 24960; 24961; 24970; 24971; 24980; 24981; 24990; 24991; 25002; 25003; 25010; 25012; 25013; 25021; 25022; 25023; 25030; 25031; 25032; 25033; 25040; 25041; 25042; 25043; 25050; 25051; 25052; 25053; 25060; 25061; 25062; 25063; 25070; 25071; 25072; 25073; 25080; 25081; 25082; 25083; 25090; 25091; 25092; 25093; 3572; 36201; 36202; 36203; 36204; 36205; 36206; 36207; 36641; E0821; E0822; E0829; E08311; E08319; E083211; E083212; E083213; E083219; E08329; E083291; E083292; E083293; E083299; E083311; E083312; E083313; E083319; E083391; E083392; E083393; E083399; E083411; E083412; E083413; E083419; E083491; E083492; E083493; E083499; E083511; E083512; E083513; E083519; E083521; E083522; E083523; E083529; E083531; E083532; E083533; E083539; E083541; E083542; E083543; E083549; E083551; E083552; E083553; E083559; E083591; E083592; E083593; E083599; E0836; E0837X1; E0837X2; E0837X3; E0837X9; E0839; E0840; E0841; E0842; E0843; E0844; E0849; E0851; E0852; E0859; E08610; E08618; E08620; E08621; E08622; E08628; E08630; E08638; E08641; E08649; E0865; E0869; E088; E089; E0900; E0901; E0910; E0911; E0921; E0922; E0929; E09311; E09319; E093211; E093212; E093213; E093219; E093291; E093292; E093293; E093299; E09331; E093311; E093312; E093313; E093319; E093391; E093392; E093393; E093399; E093411; E093412; E093413; E093419; E093491; E093492; E093493; E093499; E093511; E093512; E093513; E093519; E093521; E093522; E093523; E093529; E093531; E093532; E093533; E093539; E093541; E093542; E093543; E093549; E093551; E093552; E093553; E093559; E09359; E093591; E093592; E093593; E093599; E0936; E0937X1; E0937X2; E0937X3; E0937X9; E0939; E0940; E0941; E0942; E0943; E0944; E0949; E0951; E0952; E0959; E09610; E09618; E09620; E09621; E09622; E09628; E09630; E09638; E09641; E09649; E0965; E0969; E098; E1021; E1022; E1029; E10311; E10319; E10321; E103211; E103212; E103213; E103219; E10329; E103291; E103292; E103293; E103299; E10331; E103311; E103312; E103313; E103319; E10339; E103391; E103392; E103393; E103399; E10341; E103411; E103412; E103413; E103419; E10349; E103491; E103492; E103493; E103499; E10351; E103511; E103512; E103513; E103519; E103521; E103522; E103523; E103529; E103531; E103532; E103533; E103539; E103541; E103542; E103543; E103549; E103551; E103552; E103553; E103559; E10359; E103591; E103592; E103593; E103599; E1036; E1037X1; E1037X2; E1037X3; E1037X9; E1039; E1040; E1041; E1042; E1043; E1044; E1049; E1051; E1052; E1059; E10610; E10618; E10620; E10621; E10622; E10628; E10630; E10638; E10641; E10649; E1065; E1069; E108; E1121; E1122; E1129; E11311; E11319; E11321; E113211; E113212; E113213; E113219; E11329; E113291; E113292; E113293; E113299; E11331; E113311; E113312; E113313; E113319; E11339; E113391; E113392; E113393; E113399; E11341; E113411; E113412; E113413; E113419; E11349; E113491; E113492; E113493; E113499; E11351; E113511; E113512; E113513; E113519; E113521; E113522; E113523; E113529; E113531; E113532; E113533; E113539; E113541; E113542; E113543; E113549; E113551; E113552; E113553; E113559; E11359; E113591; E113592; E113593; E113599; E1136; E1137X1; E1137X2; E1137X3; E1137X9; E1139; E1140; E1141; E1142; E1143; E1144; E1149; E1151; E1152; E1159; E11610; E11618; E11620; E11621; E11622; E11628; E11630; E11638; E11641; E11649; E1165; E1169; E118; E1321; E1322; E1329; E13311; E13319; E13321; E133211; E133212; E133213; E133219; E13329; E133291; E133292; E133293; E133299; E13331; E133311; E133312; E133313; E133319; E13339; E133391; E133392; E133393; E133399; E13341; E133411; E133412; E133413; E133419; E13349; E133491; E133492; E133493; E133499; E13351; E133511; E133512; E133513; E133519; E133521; E133522; E133523; E133529; E133531; E133532; E133533; E133539; E133541; E133542; E133543; E133549; E133551; E133552; E133553; E133559; E133591; E133592; E133593; E133599; E1336; E1337X1; E1337X2; E1337X3; E1337X9; E1339; E1340; E1341; E1342; E1343; E1344; E1349; E1351; E1352; E1359; E13610; E13618; E13620; E13621; E13622; E13628; E13630; E13638; E13641; E13649; E1365; E1369; E138 |
| Myocardial Infarction | I2101; I2102; I2109; I2111; I2119; I2121; I2129; I213; I214; I219 |
| Coronary Artery Disease | 41000; 41001; 41002; 41010; 41011; 41012; 41020; 41021; 41022; 41030; 41031; 41032; 41040; 41041; 41042; 41050; 41051; 41052; 41060; 41061; 41062; 41070; 41071; 41072; 41080; 41081; 41082; 41090; 41091; 41092; 4110; 4111; 41181; 41189; 412; 41400; 41401; 41402; 41403; 41404; 41405; 41406; 41407; 41410; 41411; 41412; 41419; 4142; 4143; 4144; 4148; 4149; 4292; 42979; I200; I2101; I2102; I2109; I2111; I2119; I2121; I2129; I213; I214; I219; I21A1; I21A9; I220; I221; I222; I228; I229; I230; I231; I232; I233; I234; I235; I236; I237; I238; I240; I241; I248; I249; I2510; I25111; I25118; I25119; I252; I253; I2541; I2542; I255; I256; I25700; I25701; I25708; I25709; I25710; I25711; I25718; I25719; I25720; I25721; I25728; I25729; I25730; I25731; I25738; I25739; I25750; I25751; I25758; I25759; I25760; I25761; I25768; I25769; I25790; I25791; I25798; I25799; I25810; I25811; I25812; I2582; I2583; I2584; I2589; I259 |
| Cerebrovascular Disease or  Ischemic Stroke | 1671; 43301; 43311; 43321; 43331; 43381; 43391; 43401; 43411; 43491; I6300; I63011; I63012; I63013; I63019; I6302; I63031; I63032; I63033; I63039; I6309; I6310; I63111; I63112; I63113; I63119; I6312; I63131; I63132; I63133; I63139; I6319; I6320; I63211; I63212; I63213; I63219; I6322; I63231; I63232; I63233; I63239; I6329; I6330; I63311; I63312; I63313; I63319; I63321; I63322; I63323; I63329; I63331; I63332; I63333; I63339; I63341; I63342; I63343; I63349; I6339; I6340; I63411; I63412; I63413; I63419; I63421; I63422; I63423; I63429; I63431; I63432; I63433; I63439; I63441; I63442; I63443; I63449; I6349; I6350; I63511; I63512; I63513; I63519; I63521; I63522; I63523; I63529; I63531; I63532; I63533; I63539; I63541; I63542; I63543; I63549; I6359; I636; I638; I6381; I6389; I639 |
| Transient Ischemic Attack | 4350; 4351;4352;4353;4358;4359;4377; G450; G451; G452; G453; G454; G458; G459 |
| Peripheral Arterial Disease | 34839; 43310; 4400; 4401; 44020; 44021; 44022; 44023; 44024; 44029; 44030; 44031; 44032; 4404; 4408; 4409; 44100; 44101; 44102; 44103; 4411; 4412; 4413; 4414; 4415; 4416; 4417; 4419; 4420; 4421; 4422; 4423; 44281; 44282; 44283; 44284; 44289; 4429; 4431; 44321; 44322; 44323; 44324; 44329; 44382; 44389; 4439; 44401; 44409; 4441; 44421; 44422; 44481; 44489; 4449; 4470; 4471; 4472; 4473; 4474; 4475; 4476; 44770; 44771; 44772; 44773; 4478; 4479; 5571; 5579; I700; I701; I70201; I70202; I70203; I70208; I70209; I70211; I70212; I70213; I70218; I70219; I70221; I70222; I70223; I70228; I70229; I70231; I70232; I70233; I70234; I70235; I70238; I70239; I70241; I70242; I70243; I70244; I70245; I70248; I70249; I7025; I70261; I70262; I70263; I70268; I70269; I70291; I70292; I70293; I70298; I70299; I70301; I70302; I70303; I70308; I70309; I70311; I70312; I70313; I70318; I70319; I70321; I70322; I70323; I70328; I70329; I70331; I70332; I70333; I70334; I70335; I70338; I70339; I70341; I70342; I70343; I70344; I70345; I70348; I70349; I7035; I70361; I70362; I70363; I70368; I70369; I70391; I70392; I70393; I70398; I70399; I70401; I70402; I70403; I70408; I70409; I70411; I70412; I70413; I70418; I70419; I70421; I70422; I70423; I70428; I70429; I70431; I70432; I70433; I70434; I70435; I70438; I70439; I70441; I70442; I70443; I70444; I70445; I70448; I70449; I7045; I70461; I70462; I70463; I70468; I70469; I70491; I70492; I70493; I70498; I70499; I70501; I70502; I70503; I70508; I70509; I70511; I70512; I70513; I70518; I70519; I70521; I70522; I70523; I70528; I70529; I70531; I70532; I70533; I70534; I70535; I70538; I70539; I70541; I70542; I70543; I70544; I70545; I70548; I70549; I7055; I70561; I70562; I70563; I70568; I70569; I70591; I70592; I70593; I70598; I70599; I70601; I70602; I70603; I70608; I70609; I70611; I70612; I70613; I70618; I70619; I70621; I70622; I70623; I70628; I70629; I70631; I70632; I70633; I70634; I70635; I70638; I70639; I70641; I70642; I70643; I70644; I70645; I70648; I70649; I7065; I70661; I70662; I70663; I70668; I70669; I70691; I70692; I70693; I70698; I70699; I70701; I70702; I70703; I70708; I70709; I70711; I70712; I70713; I70718; I70719; I70721; I70722; I70723; I70728; I70729; I70731; I70732; I70733; I70734; I70735; I70738; I70739; I70741; I70742; I70743; I70744; I70745; I70748; I70749; I7075; I70761; I70762; I70763; I70768; I70769; I70791; I70792; I70793; I70798; I70799; I708; I7090; I7091; I7092; I7100; I7101; I71010; I71011; I71012; I71019; I7102; I7103; I711; I7110; I7111; I7112; I7113; I712; I7120; I7121; I7122; I7123; I713; I7130; I7131; I7132; I7133; I714; I7140; I7141; I7142; I7143; I715; I7150; I7151; I7152; I716; I7160; I7161; I7162; I718; I719; I720; I721; I722; I723; I724; I725; I726; I728; I729; I7301; I731; I7381; I7389; I739; I7401; I7409; I7410; I7411; I7419; I742; I743; I744; I745; I748; I749; I770; I771; I772; I773; I774; I775; I776; I7770; I7771; I7772; I7773; I7774; I7775; I7776; I7777; I7779; I77810; I77811; I77812; I77819; I7782; I7789; I779; K551; K558; K559; M318; M319 |
| Heart Failure | I0981; I110; I130; I132; I501; I5020; I5021; I5022; I5023; I5030; I5031; I5032; I5033; I5040; I5041; I5042; I5043; I50810; I50811; I50812; I50813; I50814; I5082; I5083; I5084; I5089; I509; I5181; I97130; I97131; O29121; O29122; O29123; O29129; R570; Z95811; Z95812 |
| Moderate to Severe Chronic Kidney Disease | N183; N1830; N1831; N1832; N189; N184; N185; N186; N189; Z4901; Z4902; Z4931; Z4932; Z9115; Z940; Z992; 5853; 5854; 5855 |
| Depression | F3130; F3131; F3132; F314; F315; F320; F321; F322; F323; F324; F325; F3281; F3289; F329; F330; F331; F332; F333; F3340; F3341; F3342; F338; F3341; F3342; F338; F339; F412; F432 |
| Dementia | F0150; F0151; F01511; F01518; F0152; F0153; F0154; F01A0; F01A11; F01A18; F01A2; F01A3; F01A4; F01B0; F01B11; F01B18; F01B2; F01B3; F01B4; F01C0; F01C11; F01C18; F01C2; F01C3; F01C4; F0280; F0281; F02811; F02818; F0282; F0283; F0284; F02A0; F02A11; F02A18; F02A2; F02A3; F02A4; F02B0; F02B11; F02B18; F02B2; F02B3; F02B4; F02C0; F02C11; F02C18; F02C2; F02C3; F02C4; F0390; F0391; F03911; F03918; F0392; F0393; F0394; F03A0; F03A11; F03A18; F03A2; F03A3; F03A4; F03B0; F03B11; F03B18; F03B2; F03B3; F03B4; F03C0; F03C11; F03C18; F03C2; F03C3; F03C4; F0670; F0671; G300; G301; G308; G309; G3101; G3109; G311; G312; G3181; G3182; G3183; G3185; G3189; G319 |
| Obesity | E6601; E6609; E661; E662; E668; E669; O99210; O99211; O99212; O99213; O99214; O99215; R939; Z6830; Z6831; Z6832; Z6833; Z6834; Z6835; Z6836; Z6837; Z6838; Z6839; Z6841; Z6842; Z6843; Z6844; Z6845; Z6854 |
| Atrial Fibrillation | 42731; 42732; I480; I481; I4811; I4819; I482; I4820; I4821; I483; I484; I4891; I4892 |
| Hyperlipidemia | 2720; 2721; 2722; 2723; 2724; 2725; 2726; 2727; 2728; 2729; E780; E7800; E7801; E781; E782; E783; E784; E7841; E7849; E785; E786; E7870; E7871; E7872; E7879; E7881; E7889; E789 |
| Mild Liver disease | 701;7030;7031;7032;7033;7051;7052;7053;7054;7059;7070;709;9162;952;953;5710;5711;5712;5713;57140;57141;57142;57149;5715;5716;5718;5719;5720;5728;5730;5731;5732;5733;5734;5735;5738;5739;A5145;A5274;B180;B181;B182;B188;B189;B1910;B1920;B199;B251;B581;K700;K7010;K7011;K702;K7030;K7031;K709;K713;K714;K7150;K7151;K716;K717;K718;K730;K731;K732;K738;K739;K740;K7400;K7401;K7402;K741;K742;K743;K744;K745;K7460;K7469;K751;K752;K753;K754;K7581;K7589;K759;K760;K761;K762;K763;K764;K7681;K7682;K7689;K769;K77 |
| Severe Liver disease | 700; 7020; 7021; 7022; 7023; 7041; 7043; 7044; 7049; 706; 7071; 4560; 4561; 45620; 45621; 4568; 570; 5721; 5722; 5723; 5724; 99749; B190; B1911; B1921; I8500; I8501; I8510; I8511; I864; K7040; K7041; K7210; K7211; K7290; K7291; K765; K766; K767; K9182; V427; Z944 |
| Hemodialysis | V4511; Z992 |
| Valvular heart disease | 3940; 3941; 3942; 3949; 3950; 3951; 3952; 3959; 3960; 3961; 3962; 3963; 3968; 3969; 3970; 3971; 3979; 39890; 39891; 39899; 4240; 4241; 4242; 4243; 74600; 74601; 74602; 74609; 7461; 7462; 7463; 7464; 7465; 7466; 7467; 99602; 99671; I050; I051; I052; I058; I059; I060; I061; I062; I068; I069; I070; I071; I072; I078; I079; I080; I081; I082; I083; I088; I089; I091; I0981; I0989; I099; I340; I341; I342; I348; I349; I350; I351; I352; I358; I359; I360; I361; I362; I368; I369; I370; I371; I372; I378; I379; Q220; Q221; Q222; Q223; Q224; Q225; Q226; Q228; Q229; Q230; Q231; Q232; Q233; Q234; Q238; Q239; T8201XA; T8201XD; T8201XS; T8202XA; T8202XD; T8202XS; T8203XA; T8203XD; T8203XS; T8209XA; T8209XD; T8209XS; T82221A; T82221D; T82221S; T82222A; T82222D; T82222S; T82223A; T82223D; T82223S; T82228A; T82228D; T82228S; T826XXA; T826XXD; T826XXS; V422; V433; Z952; Z953; Z954 |
| Asthma | 49300; 49301; 49302; 49310; 49311; 49312; 49320; 49321; 49322; 49381; 49382; 49390; 49391; 49392; J4520; J4521; J4522; J4530; J4531; J4532; J4540; J4541; J4542; J4550; J4551; J4552; J45901; J45902; J45909; J45990; J45991; J45998; |
| COPD | 490; 4910; 4911; 4912; 49120; 49121; 49122; 4918; 4919; 4920; 4928; 494; 4940; 4941; 496; J40; J410; J411; J418; J42; J430; J431; J432; J438; J439; J440; J441; J449; J470; J471; J479 |
| Hyperlipidemia | 2720;2721;2722;2723;2724;2725;2726;2727;2728;2729; E780; E7800; E7801; E781; E782; E783; E784; E7841; E7849; E785; E786; E7870; E7871; E7872; E7879; E7881; E7889; E789 |
| Hypertension | 4010; 4011; 4019; 40200; 40201; 40210; 40211; 40290; 40291; 40300; 40301; 40310; 40311; 40390; 40391; 40400; 40401; 40402; 40403; 40410; 40411; 40412; 40413; 40490; 40491; 40492; 40493; 40501; 40509; 40511; 40519; 40591; 40599; I10; I110; I119; I120; I129; I130; I1310; I1311; I132; I150; I151; I152; I158; I159; I160; I161; I169 |
|  | **LOINC codes** |
| LDL | 13457-7;18261-8;18262-6;2089-1;2090-9;35198-1;49132-4;55440-2;96259-7;96597-0 |
| Blood Urea Nitrogen | 3094-0;11064-3;11065-0;12961-9;12962-7;12964-3;12965-0;12966-8;20977-5;6299-2;3091-6;35234-4;42571-0;72270-2;93975-1;94003-1;94012-2 |
| Hemoglobin | 17855-8;17856-6;43150-2;4548-4;4549-2;54039-3;55454-3;59261-8;62388-4;67761-7;71875-9;74246-0;75862-3;86910-7;96070-8;96595-4;62853-7;62854-5 |
| Creatinine | 11041-1; 11042-9; 16188-5; 16189-3; 21232-4; 2160-0; 35203-9; 38483-4; 39955-0; 39956-8; 39957-6; 39958-4; 39959-2; 39960-0; 39961-8; 39962-6; 39963-4; 39964-2; 39965-9; 39966-7; 39967-5; 39968-3; 39969-1; 39970-9; 39971-7; 39972-5; 39973-3; 39974-1; 39975-8; 39976-6; 40248-7; 40249-5; 40250-3; 40251-1; 40252-9; 40253-7; 40254-5; 40255-2; 40256-0; 40257-8; 40258-6; 44784-7; 72271-0; 93999-1; 94008-0; 96590-5 |
| hsCRP | 30522-7; 71426-1; 76486-0 |
|  | **Procedure codes** |
| Peripheral Arterial Revascularization | 0238T; 35381; 35450; 35452; 35454; 35456; 35459; 35470; 35472; 35473; 35474; 35481; 35482; 35483; 35485; 35491; 35492; 35493; 35495; 35521; 35533; 35537; 35538; 35539; 35540; 35541; 35546; 35548; 35549; 35551; 35556; 35558; 35563; 35565; 35566; 35571; 35582; 35583; 35585; 35587; 35621; 35623; 35637; 35638; 35641; 35646; 35647; 35651; 35654; 35656; 35661; 35663; 35665; 35666; 35671; 35879; 35881; 35883; 35884; 36140; 36200; 36245; 36246; 36247; 36248; 37184; 37185; 37186; 37205; 37206; 37207; 37208; 37220; 37221; 37222; 37223; 37224; 37225; 37226; 37227; 37228; 37229; 37230; 37231; 37232; 37233; 37234; 37235; 37236; 37237; 37238; 37239; 0045; 0046; 0047; 0048; 0055; 0060; 1756; 3808; 3818; 3838; 3848; 3925; 3929; 3949; 3950; 3956; 3957; 3958; 3990; 047C04Z; 047C0DZ; 047C0ZZ; 047C34Z; 047C3DZ; 047C3ZZ; 047C44Z; 047C4DZ; 047C4ZZ; 047D04Z; 047D0DZ; 047D0ZZ; 047D34Z; 047D3DZ; 047D3ZZ; 047D44Z; 047D4DZ; 047D4ZZ; 047E04Z; 047E0DZ; 047E0ZZ; 047E34Z; 047E3DZ; 047E3ZZ; 047E44Z; 047E4DZ; 047E4ZZ; 047F04Z; 047F0DZ; 047F0ZZ; 047F34Z; 047F3DZ; 047F3ZZ; 047F44Z; 047F4DZ; 047F4ZZ; 047H04Z; 047H0DZ; 047H0ZZ; 047H34Z; 047H3DZ; 047H3ZZ; 047H44Z; 047H4DZ; 047H4ZZ; 047J04Z; 047J0DZ; 047J0ZZ; 047J34Z; 047J3DZ; 047J3ZZ; 047J44Z; 047J4DZ; 047J4ZZ; 047K041; 047K04Z; 047K0D1; 047K0DZ; 047K0Z1; 047K0ZZ; 047K341; 047K34Z; 047K3D1; 047K3DZ; 047K3Z1; 047K3ZZ; 047K441; 047K44Z; 047K4D1; 047K4DZ; 047K4Z1; 047K4ZZ; 047L041; 047L04Z; 047L0D1; 047L0DZ; 047L0Z1; 047L0ZZ; 047L341; 047L34Z; 047L3D1; 047L3DZ; 047L3Z1; 047L3ZZ; 047L441; 047L44Z; 047L4D1; 047L4DZ; 047L4Z1; 047L4ZZ; 047M041; 047M04Z; 047M0D1; 047M0DZ; 047M0Z1; 047M0ZZ; 047M341; 047M34Z; 047M3D1; 047M3DZ; 047M3Z1; 047M3ZZ; 047M441; 047M44Z; 047M4D1; 047M4DZ; 047M4Z1; 047M4ZZ; 047N041; 047N04Z; 047N0D1; 047N0DZ; 047N0Z1; 047N0ZZ; 047N341; 047N34Z; 047N3D1; 047N3DZ; 047N3Z1; 047N3ZZ; 047N441; 047N44Z; 047N4D1; 047N4DZ; 047N4Z1; 047N4ZZ; 047P04Z; 047P0DZ; 047P0ZZ; 047P34Z; 047P3DZ; 047P3ZZ; 047P44Z; 047P4DZ; 047P4ZZ; 047Q04Z; 047Q0DZ; 047Q0ZZ; 047Q34Z; 047Q3DZ; 047Q3ZZ; 047Q44Z; 047Q4DZ; 047Q4ZZ; 047R04Z; 047R0DZ; 047R0ZZ; 047R34Z; 047R3DZ; 047R3ZZ; 047R44Z; 047R4DZ; 047R4ZZ; 047S04Z; 047S0DZ; 047S0ZZ; 047S34Z; 047S3DZ; 047S3ZZ; 047S44Z; 047S4DZ; 047S4ZZ; 047T04Z; 047T0DZ; 047T0ZZ; 047T34Z; 047T3DZ; 047T3ZZ; 047T44Z; 047T4DZ; 047T4ZZ; 047U04Z; 047U0DZ; 047U0ZZ; 047U34Z; 047U3DZ; 047U3ZZ; 047U44Z; 047U4DZ; 047U4ZZ; 047V04Z; 047V0DZ; 047V0ZZ; 047V34Z; 047V3DZ; 047V3ZZ; 047V44Z; 047V4DZ; 047V4ZZ; 047W04Z; 047W0DZ; 047W0ZZ; 047W34Z; 047W3DZ; 047W3ZZ; 047W44Z; 047W4DZ; 047W4ZZ; 031209B; 031209C; 03120A6; 03120A7; 03120A8; 03120A9; 03120AB; 03120AC; 03120J6; 03120J7; 03120J8; 03120J9; 03120JB; 03120JC; 03120K6; 03120K7; 03120K8; 03120K9; 03120KB; 03120KC; 03120Z6; 03120Z7; 03120Z8; 03120Z9; 03120ZB; 03120ZC; 031309B; 031309C; 03130A6; 03130A7; 03130A8; 03130A9; 03130AB; 03130AC; 03130J6; 03130J7; 03130J8; 03130J9; 03130JB; 03130JC; 03130K6; 03130K7; 03130K8; 03130K9; 03130KB; 03130KC; 041009B; 041009C; 041009D; 041009F; 041009G; 041009H; 041009J; 041009K; 041009Q; 041009R; 04100A6; 04100A7; 04100A8; 04100A9; 04100AB; 04100AC; 04100AD; 04100AF; 04100AG; 04100AH; 04100AJ; 04100AK; 04100AQ; 04100AR; 04100J6; 04100J7; 04100J8; 04100J9; 04100JB; 04100JC; 04100JD; 04100JF; 04100JG; 04100JH; 04100JJ; 04100JK; 04100JQ; 04100JR; 04100K6; 04100K7; 04100K8; 04100K9; 04100KB; 04100KC; 04100KD; 04100KF; 04100KG; 04100KH; 04100KJ; 04100KK; 04100KQ; 04100KR; 04100Z6; 04100Z7; 04100Z8; 04100Z9; 04100ZB; 04100ZC; 04100ZD; 04100ZF; 04100ZG; 04100ZH; 04100ZJ; 04100ZK; 04100ZQ; 04100ZR; 041049B; 041049C; 041049D; 041049F; 041049G; 041049H; 041049J; 041049K; 041049Q; 041049R; 04104A6; 04104A7; 04104A8; 04104A9; 04104AB; 04104AC; 04104AD; 04104AF; 04104AG; 04104AH; 04104AJ; 04104AK; 04104AQ; 04104AR; 04104J6; 04104J7; 04104J8; 04104J9; 04104JB; 04104JC; 04104JD; 04104JF; 04104JG; 04104JH; 04104JJ; 04104JK; 04104JQ; 04104JR; 04104K6; 04104K7; 04104K8; 04104K9; 04104KB; 04104KC; 04104KD; 04104KF; 04104KG; 04104KH; 04104KJ; 04104KK; 04104KQ; 04104KR; 04104Z6; 04104Z7; 04104Z8; 04104Z9; 04104ZB; 04104ZC; 04104ZD; 04104ZF; 04104ZG; 04104ZH; 04104ZJ; 04104ZK; 04104ZQ; 04104ZR; 041C09H; 041C09J; 041C09K; 041C0AH; 041C0AJ; 041C0AK; 041C0JH; 041C0JJ; 041C0JK; 041C0KH; 041C0KJ; 041C0KK; 041C0ZH; 041C0ZJ; 041C0ZK; 041C49H; 041C49J; 041C49K; 041C4AH; 041C4AJ; 041C4AK; 041C4JH; 041C4JJ; 041C4JK; 041C4KH; 041C4KJ; 041C4KK; 041C4ZH; 041C4ZJ; 041C4ZK; 041D09H; 041D09J; 041D09K; 041D0AH; 041D0AJ; 041D0AK; 041D0JH; 041D0JJ; 041D0JK; 041D0KH; 041D0KJ; 041D0KK; 041D0ZH; 041D0ZJ; 041D0ZK; 041D49H; 041D49J; 041D49K; 041D4AH; 041D4AJ; 041D4AK; 041D4JH; 041D4JJ; 041D4JK; 041D4KH; 041D4KJ; 041D4KK; 041D4ZH; 041D4ZJ; 041D4ZK; 041E09H; 041E09J; 041E09K; 041E0AH; 041E0AJ; 041E0AK; 041E0JH; 041E0JJ; 041E0JK; 041E0KH; 041E0KJ; 041E0KK; 041E0ZH; 041E0ZJ; 041E0ZK; 041E49H; 041E49J; 041E49K; 041E4AH; 041E4AJ; 041E4AK; 041E4JH; 041E4JJ; 041E4JK; 041E4KH; 041E4KJ; 041E4KK; 041E4ZH; 041E4ZJ; 041E4ZK; 041F09H; 041F09J; 041F09K; 041F0AH; 041F0AJ; 041F0AK; 041F0JH; 041F0JJ; 041F0JK; 041F0KH; 041F0KJ; 041F0KK; 041F0ZH; 041F0ZJ; 041F0ZK; 041F49H; 041F49J; 041F49K; 041F4AH; 041F4AJ; 041F4AK; 041F4JH; 041F4JJ; 041F4JK; 041F4KH; 041F4KJ; 041F4KK; 041F4ZH; 041F4ZJ; 041F4ZK; 041H09H; 041H09J; 041H09K; 041H0AH; 041H0AJ; 041H0AK; 041H0JH; 041H0JJ; 041H0JK; 041H0KH; 041H0KJ; 041H0KK; 041H0ZH; 041H0ZJ; 041H0ZK; 041H49H; 041H49J; 041H49K; 041H4AH; 041H4AJ; 041H4AK; 041H4JH; 041H4JJ; 041H4JK; 041H4KH; 041H4KJ; 041H4KK; 041H4ZH; 041H4ZJ; 041H4ZK; 041J09H; 041J09J; 041J09K; 041J0AH; 041J0AJ; 041J0AK; 041J0JH; 041J0JJ; 041J0JK; 041J0KH; 041J0KJ; 041J0KK; 041J0ZH; 041J0ZJ; 041J0ZK; 041J49H; 041J49J; 041J49K; 041J4AH; 041J4AJ; 041J4AK; 041J4JH; 041J4JJ; 041J4JK; 041J4KH; 041J4KJ; 041J4KK; 041J4ZH; 041J4ZJ; 041J4ZK; 041K09H; 041K09J; 041K09K; 041K09L; 041K09M; 041K09N; 041K09P; 041K09Q; 041K09S; 041K0AH; 041K0AJ; 041K0AK; 041K0AL; 041K0AM; 041K0AN; 041K0AP; 041K0AQ; 041K0AS; 041K0JH; 041K0JJ; 041K0JK; 041K0JL; 041K0JM; 041K0JN; 041K0JP; 041K0JQ; 041K0JS; 041K0KH; 041K0KJ; 041K0KK; 041K0KL; 041K0KM; 041K0KN; 041K0KP; 041K0KQ; 041K0KS; 041K0ZH; 041K0ZJ; 041K0ZK; 041K0ZL; 041K0ZM; 041K0ZN; 041K0ZP; 041K0ZQ; 041K0ZS; 041K49H; 041K49J; 041K49K; 041K49L; 041K49M; 041K49N; 041K49P; 041K49Q; 041K49S; 041K4AH; 041K4AJ; 041K4AK; 041K4AL; 041K4AM; 041K4AN; 041K4AP; 041K4AQ; 041K4AS; 041K4JH; 041K4JJ; 041K4JK; 041K4JL; 041K4JM; 041K4JN; 041K4JP; 041K4JQ; 041K4JS; 041K4KH; 041K4KJ; 041K4KK; 041K4KL; 041K4KM; 041K4KN; 041K4KP; 041K4KQ; 041K4KS; 041K4ZH; 041K4ZJ; 041K4ZK; 041K4ZL; 041K4ZM; 041K4ZN; 041K4ZP; 041K4ZQ; 041K4ZS; 041L09H; 041L09J; 041L09K; 041L09L; 041L09M; 041L09N; 041L09P; 041L09Q; 041L09S; 041L0AH; 041L0AJ; 041L0AK; 041L0AL; 041L0AM; 041L0AN; 041L0AP; 041L0AQ; 041L0AS; 041L0JH; 041L0JJ; 041L0JK; 041L0JL; 041L0JM; 041L0JN; 041L0JP; 041L0JQ; 041L0JS; 041L0KH; 041L0KJ; 041L0KK; 041L0KL; 041L0KM; 041L0KN; 041L0KP; 041L0KQ; 041L0KS; 041L0ZH; 041L0ZJ; 041L0ZK; 041L0ZL; 041L0ZM; 041L0ZN; 041L0ZP; 041L0ZQ; 041L0ZS; 041L49H; 041L49J; 041L49K; 041L49L; 041L49M; 041L49N; 041L49P; 041L49Q; 041L49S; 041L4AH; 041L4AJ; 041L4AK; 041L4AL; 041L4AM; 041L4AN; 041L4AP; 041L4AQ; 041L4AS; 041L4JH; 041L4JJ; 041L4JK; 041L4JL; 041L4JM; 041L4JN; 041L4JP; 041L4JQ; 041L4JS; 041L4KH; 041L4KJ; 041L4KK; 041L4KL; 041L4KM; 041L4KN; 041L4KP; 041L4KQ; 041L4KS; 041L4ZH; 041L4ZJ; 041L4ZK; 041L4ZL; 041L4ZM; 041L4ZN; 041L4ZP; 041L4ZQ; 041L4ZS; 041M09L; 041M09M; 041M09P; 041M09Q; 041M09S; 041M0AL; 041M0AM; 041M0AP; 041M0AQ; 041M0AS; 041M0JL; 041M0JM; 041M0JP; 041M0JQ; 041M0JS; 041M0KL; 041M0KM; 041M0KP; 041M0KQ; 041M0KS; 041M0ZL; 041M0ZM; 041M0ZP; 041M0ZQ; 041M0ZS; 041M49L; 041M49M; 041M49P; 041M49Q; 041M49S; 041M4AL; 041M4AM; 041M4AP; 041M4AQ; 041M4AS; 041M4JL; 041M4JM; 041M4JP; 041M4JQ; 041M4JS; 041M4KL; 041M4KM; 041M4KP; 041M4KQ; 041M4KS; 041M4ZL; 041M4ZM; 041M4ZP; 041M4ZQ; 041M4ZS; 041N09L; 041N09M; 041N09P; 041N09Q; 041N09S; 041N0AL; 041N0AM; 041N0AP; 041N0AQ; 041N0AS; 041N0JL; 041N0JM; 041N0JP; 041N0JQ; 041N0JS; 041N0KL; 041N0KM; 041N0KP; 041N0KQ; 041N0KS; 041N0ZL; 041N0ZM; 041N0ZP; 041N0ZQ; 041N0ZS; 041N49L; 041N49M; 041N49P; 041N49Q; 041N49S; 041N4AL; 041N4AM; 041N4AP; 041N4AQ; 041N4AS; 041N4JL; 041N4JM; 041N4JP; 041N4JQ; 041N4JS; 041N4KL; 041N4KM; 041N4KP; 041N4KQ; 041N4KS; 041N4ZL; 041N4ZM; 041N4ZP; 041N4ZQ; 041N4ZS; 04RK07Z; 04RK0JZ; 04RK0KZ; 04RK47Z; 04RK4JZ; 04RK4KZ; 04RL07Z; 04RL0JZ; 04RL0KZ; 04RL47Z; 04RL4JZ; 04RL4KZ; 04RM07Z; 04RM0JZ; 04RM0KZ; 04RM47Z; 04RM4JZ; 04RM4KZ; 04RN07Z; 04RN0JZ; 04RN0KZ; 04RN47Z; 04RN4JZ; 04RN4KZ; 04RP07Z; 04RP0JZ; 04RP0KZ; 04RP47Z; 04RP4JZ; 04RP4KZ; 04RQ07Z; 04RQ0JZ; 04RQ0KZ; 04RQ47Z; 04RQ4JZ; 04RQ4KZ; 04RR07Z; 04RR0JZ; 04RR0KZ; 04RR47Z; 04RR4JZ; 04RR4KZ; 04RS07Z; 04RS0JZ; 04RS0KZ; 04RS47Z; 04RS4JZ; 04RS4KZ; 04RT07Z; 04RT0JZ; 04RT0KZ; 04RT47Z; 04RT4JZ; 04RT4KZ; 04RU07Z; 04RU0JZ; 04RU0KZ; 04RU47Z; 04RU4JZ; 04RU4KZ; 04RV07Z; 04RV0JZ; 04RV0KZ; 04RV47Z; 04RV4JZ; 04RV4KZ; 04RW07Z; 04RW0JZ; 04RW0KZ; 04RW47Z; 04RW4JZ; 04RW4KZ; 04RY07Z; 04RY0JZ; 04RY0KZ; 04RY47Z; 04RY4JZ; 04RY4KZ; 04UC07Z; 04UC0JZ; 04UC0KZ; 04UC37Z; 04UC3JZ; 04UC3KZ; 04UC47Z; 04UC4JZ; 04UC4KZ; 04UD07Z; 04UD0JZ; 04UD0KZ; 04UD37Z; 04UD3JZ; 04UD3KZ; 04UD47Z; 04UD4JZ; 04UD4KZ; 04UE07Z; 04UE0JZ; 04UE0KZ; 04UE37Z; 04UE3JZ; 04UE3KZ; 04UE47Z; 04UE4JZ; 04UE4KZ; 04UF07Z; 04UF0JZ; 04UF0KZ; 04UF37Z; 04UF3JZ; 04UF3KZ; 04UF47Z; 04UF4JZ; 04UF4KZ; 04UH07Z; 04UH0JZ; 04UH0KZ; 04UH37Z; 04UH3JZ; 04UH3KZ; 04UH47Z; 04UH4JZ; 04UH4KZ; 04UJ07Z; 04UJ0JZ; 04UJ0KZ; 04UJ37Z; 04UJ3JZ; 04UJ3KZ; 04UJ47Z; 04UJ4JZ; 04UJ4KZ; 04UK07Z; 04UK0JZ; 04UK0KZ; 04UK37Z; 04UK3JZ; 04UK3KZ; 04UK47Z; 04UK4JZ; 04UK4KZ; 04UL07Z; 04UL0JZ; 04UL0KZ; 04UL37Z; 04UL3JZ; 04UL3KZ; 04UL47Z; 04UL4JZ; 04UL4KZ; 04UM07Z; 04UM0JZ; 04UM0KZ; 04UM37Z; 04UM3JZ; 04UM3KZ; 04UM47Z; 04UM4JZ; 04UM4KZ; 04UN07Z; 04UN0JZ; 04UN0KZ; 04UN37Z; 04UN3JZ; 04UN3KZ; 04UN47Z; 04UN4JZ; 04UN4KZ; 04UP07Z; 04UP0JZ; 04UP0KZ; 04UP37Z; 04UP3JZ; 04UP3KZ; 04UP47Z; 04UP4JZ; 04UP4KZ; 04UQ07Z; 04UQ0JZ; 04UQ0KZ; 04UQ37Z; 04UQ3JZ; 04UQ3KZ; 04UQ47Z; 04UQ4JZ; 04UQ4KZ; 04UR07Z; 04UR0JZ; 04UR0KZ; 04UR37Z; 04UR3JZ; 04UR3KZ; 04UR47Z; 04UR4JZ; 04UR4KZ; 04US07Z; 04US0JZ; 04US0KZ; 04US37Z; 04US3JZ; 04US3KZ; 04US47Z; 04US4JZ; 04US4KZ; 04UT07Z; 04UT0JZ; 04UT0KZ; 04UT37Z; 04UT3JZ; 04UT3KZ; 04UT47Z; 04UT4JZ; 04UT4KZ; 04UU07Z; 04UU0JZ; 04UU0KZ; 04UU37Z; 04UU3JZ; 04UU3KZ; 04UU47Z; 04UU4JZ; 04UU4KZ; 04UV07Z; 04UV0JZ; 04UV0KZ; 04UV37Z; 04UV3JZ; 04UV3KZ; 04UV47Z; 04UV4JZ; 04UV4KZ; 04UW07Z; 04UW0JZ; 04UW0KZ; 04UW37Z; 04UW3JZ; 04UW3KZ; 04UW47Z; 04UW4JZ; 04UW4KZ; 04UY07Z; 04UY0JZ; 04UY0KZ; 04UY37Z; 04UY3JZ; 04UY3KZ; 04UY47Z; 04UY4JZ; 04UY4KZ |

# **Supplement Table 2. Comparison of Patient Characteristics Excluded Due to Comorbidities**

|  | Included | Excluded | p-value |
| --- | --- | --- | --- |
|  | N=94,166 | N=8,173 |  |
| Age | 72.7 (9.1) | 70.2 (9.4) | <0.001 |
| Female Sex | 3,613 ( 3.8%) | 224 ( 2.7%) | <0.001 |
| Acute Myocardial infarction | 5,382 (5.7%) | 1,854 (22.7%) | <0.001 |
| Cancer, Metastatic | 0 (0.0%) | 2,015 (24.7%) | <0.001 |
| Heart Failure | 33,982 (36.1) | 4,089 (50.0%) | <0.001 |
| HIV | 0 (0.0%) | 689 ( 8.4%) | <0.001 |
| Ischemic Stroke | 30983 (32.9) | 1,464 (17.9%) | <0.001 |
| Liver Disease, Moderate or Severe | 1833 (1.9) | 2,015 (24.7%) | <0.001 |
| Osteomyelitis, Chronic | 0 (0.0%) | 1,710 (20.9%) | <0.001 |
| Chemotherapy | 0 (0.0%) | 1,208 (14.8%) | <0.001 |
| Chronic Kidney Disease Stage 2 | 4,283 ( 4.5%) | 274 (3.4%) | <0.001 |
| Chronic Kidney Disease Stage 3 | 82,226 (87.3%) | 4,491 (55.0%) |  |
| Chronic Kidney Disease Stage 4 | 7,657 ( 8.1%) | 473 (5.8%) |  |
| Chronic Kidney Disease Stage 5 | 0 (0.0%) | 2,935 (35.9%) |  |

# **Supplement Table 3. Comparison of Patient Characteristics with and Without hsCRP Testing**

|  | Overall | No hsCRP Test | hsCRP Test | Standardized Mean Differences |
| --- | --- | --- | --- | --- |
|  | n=354,067 | n=259,901 | n=94,166 |  |
| **Demographic** |  |  |  |  |
| Age | 72.39 (6.97) | 72.29 (5.99) | 72.66 (9.15) | 0.048 |
| Female Sex | 20843 (5.9) | 17230 (6.6) | 3613 (3.8) | 0.126 |
| *Race* |  |  |  | 0.060 |
| Asian | 1979 (0.6) | 1370 (0.5) | 609 (0.6) |  |
| Indian/Native | 2762 (0.8) | 1934 (0.7) | 828 (0.9) |  |
| Black | 61817 (17.5) | 44238 (17.0) | 17579 (18.7) |  |
| Pacific Islander | 3286 (0.9) | 2412 (0.9) | 874 (0.9) |  |
| White | 261078 (73.7) | 193431 (74.4) | 67647 (71.8) |  |
| Missing | 23145 (6.5) | 16516 (6.4) | 6629 (7.0) |  |
| *Ethnicity* |  |  |  | 0.141 |
| Non-Hispanic | 321240 (90.7) | 237863 (91.5) | 83377 (88.5) |  |
| Hispanic | 16146 (4.6) | 9670 (3.7) | 6476 (6.9) |  |
| Unknown | 16681 (4.7) | 12368 (4.8) | 4313 (4.6) |  |
| **Comorbidity** |  |  |  |  |
| Frailty | 80323 (22.7) | 56208 (21.6) | 24115 (25.6) | 0.094 |
| Coronary artery Disease | 255479 (72.2) | 184877 (71.1) | 70602 (75.0) | 0.087 |
| Cerebrovascular Disease (i.e. Stroke and TIA) | 78017 (22.0) | 47034 (18.1) | 30983 (32.9) | 0.345 |
| Peripheral Artery Disease | 152372 (43.0) | 105856 (40.7) | 46516 (49.4) | 0.175 |
| Revascularization, Coronary or Peripheral Arterial | 48569 (13.7) | 34715 (13.4) | 13854 (14.7) | 0.039 |
| Atrial Fibrillation/Atrial Flutter | 101318 (28.6) | 76455 (29.4) | 24863 (26.4) | 0.067 |
| Heart Failure | 123727 (34.9) | 89745 (34.5) | 33982 (36.1) | 0.033 |
| Valvular Heart Disease | 78852 (22.3) | 57916 (22.3) | 20936 (22.2) | 0.001 |
| Ventricular Arrhythmias | 34502 (9.7) | 27332 (10.5) | 7170 (7.6) | 0.101 |
| Myositis | 308 (0.1) | 130 (0.1) | 178 (0.2) | 0.04 |
| Multiple sclerosis | 1109 (0.3) | 735 (0.3) | 374 (0.4) | 0.02 |
| Rheumatoid Arthritis | 14212 (4.0) | 6987 (2.7) | 7225 (7.7) | 0.226 |
| Seronegative Spondyloarthropathies | 15065 (4.3) | 11733 (4.5) | 3332 (3.5) | 0.050 |
| Systemic Lupus Erythematosus | 1436 (0.4) | 796 (0.3) | 640 (0.7) | 0.053 |
| Other Systemic Connective Tissue Disorders | 933 (0.3) | 320 (0.1) | 613 (0.7) | 0.085 |
| Vasculitis | 3183 (0.9) | 1219 (0.5) | 1964 (2.1) | 0.144 |
| Asthma | 29040 (8.2) | 20293 (7.8) | 8747 (9.3) | 0.053 |
| Chronic Obstructive Pulmonary Disease | 126989 (35.9) | 90590 (34.9) | 36399 (38.7) | 0.079 |
| Diabetes Mellitus | 180977 (51.1) | 134199 (51.6) | 46778 (49.7) | 0.039 |
| Hyperlipidemia | 319431 (90.2) | 237288 (91.3) | 82143 (87.2) | 0.132 |
| Hypertension | 337731 (95.4) | 248151 (95.5) | 89580 (95.1) | 0.017 |
| Hypothyroidism | 58292 (16.5) | 43325 (16.7) | 14967 (15.9) | 0.021 |
| Liver Disease, Mild | 34807 (9.8) | 27261 (10.5) | 7546 (8.0) | 0.086 |
| Liver Disease, Moderate or Severe | 6550 (1.8) | 4717 (1.8) | 1833 (1.9) | 0.010 |
| Charlson comorbidity Index, Median (IQR) | 4 [2, 6] | 4 [2, 6] | 4 [3, 7] | 0.168 |
| 1-2 | 96585 (27.3) | 74617 (28.7) | 21968 (23.3) | 0.252 |
| 3-5 | 147773 (41.7) | 112891 (43.4) | 34882 (37.0) |  |
| >5 | 109709 (31.0) | 72393 (27.9) | 37316 (39.6) |  |
| Systolic Blood Pressure, mean (SD) | 135 (15.0) | 135 (15.4) | 135.2 (13.8) | 0.003 |
| Diastolic Blood Pressure, mean (SD) | 75 (8.3) | 75.0 (8.1) | 74 (8.8) | 0.111 |
| Hemoglobin A1c (%) | 6.6 (1.3) | 6.6 (1.3) | 6.8 (1.5) | 0.198 |
| eGFR (based on serum creatinine) | 49.7 (10.6) | 50.1 (9.7) | 48.6 (12.8) | 0.133 |
| UACR | 219 (862) | 245 (546) | 169 (1272) | 0.077 |

Abbreviation: hsCRP: high sensitivity c-reactive protein; SD: standard deviation; IQR: Interquartile range; TIA: transient ischemic stroke; eGFR: estimated glomerular filtration rate; uACR: urine albumin creatinine ratio.

# **Supplement Table 4. Comparison of Veterans with hsCRP>10 and hsCRP >2 and <10mg/L**

| Characteristics | Overall (hsCRP >2 mg/L)  (N = 70,990) | | hsCRP > 10mg/L (N=31,984) | | | hsCRP >2 and <10mg/L  (N =39,006) | P-value | | | |  |  |
| --- | --- | --- | --- | --- | --- | --- | --- | --- | --- | --- | --- | --- |
| **Demographics** |  | |  | | |  |  | | | |  |  |
| Age, Years, Mean (SD) | 72.4 (9.2) | | 72.2 (9.2) | | | 72.5 (9.1) | 0.001 | | | |  |  |
| Female, n (%) | 2693 (3.8) | | 1084 (3.4) | | | 1609 (4.1) | <0.001 | | | |  |  |
| Race, n (%) |  | |  | | |  | 0.01 | | | |  |  |
| Asian | 353 (0.5) | | 167 (0.5) | | | 186 (0.5) |  | | | |  |  |
| American Indian/Native American | 631 (0.9) | | 286 (0.9) | | | 345 (0.9) |  | | | |  |  |
| Black | 13356 (18.8) | | 6178 (19.3) | | | 7178 (18.4) |  | | | |  |  |
| Pacific Islander/Native Hawaiian | 617 (0.9) | | 280 (0.9) | | | 337 (0.9) |  | | | |  |  |
| White | 51159 (72.1) | | 22961 (71.8) | | | 28198 (72.3) |  | | | |  |  |
| Missing | 4874 (6.9) | | 2112 (6.6) | | | 2762 (7.1) |  | | | |  |  |
| Ethnicity, n (%) |  | |  | | |  | <0.001 | | | |  |  |
| Non-Hispanic/Latinx | 62878 (88.6) | | 28165 (88.1) | | | 34713 (89.0) |  | | | |  |  |
| Hispanic/Latinx | 4941 (7.0) | | 2420 (7.6) | | | 2521 (6.5) |  | | | |  |  |
| Missing | 3171 (4.5) | | 1399 (4.4) | | | 1772 (4.5) |  | | | |  |  |
| Rural designation, n (%) | 15998 (22.5) | | 6719 (21.0) | | | 9279 (23.8) | <0.001 | | | |  |  |
| Drive time to a primary care, minutes, Median (IQR) | 27.28 (24.86) | | 26.72 (25.58) | | | 27.72 (24.24) | <0.001 | | | |  |  |
| CDC SVI by quartile, n (%) |  | |  | | |  | <0.001 | | | |  |  |
| <0.25 | 10930 (15.4) | | 4679 (14.6) | | | 6251 (16.0) |  | | | |  |  |
| 0.25-<0.50 | 15756 (22.2) | | 6878 (21.5) | | | 8878 (22.8) |  | | | |  |  |
| 0.50-<0.75 | 17346 (24.4) | | 7763 (24.3) | | | 9583 (24.6) |  | | | |  |  |
| 0.75-1.00 | 18360 (25.9) | | 8557 (26.8) | | | 9803 (25.1) |  | | | |  |  |
| Missing | 8598 (12.1) | | 4107 (12.8) | | | 4491 (11.5) |  | | | |  |  |
| **Comorbidities** |  | |  | | |  |  | | | |  |  |
| *Atherosclerotic Cardiovascular Disease* |  | |  | | |  |  | | | |  |  |
| Coronary artery Disease | 53068 (74.8) | | 23821 (74.5) | | | 29247 (75.0) | 0.127 | | | |  |  |
| Cerebrovascular Disease (i.e. Stroke and TIA) | 22990 (32.4) | | 10360 (32.4) | | | 12630 (32.4) | 0.98 | | | |  |  |
| Peripheral Artery Disease | 36364 (51.2) | | 17504 (54.7) | | | 18860 (48.4) | <0.001 | | | |  |  |
| Myocardial Infarction | 3997 (5.6) | | 2090 (6.5) | | | 1907 (4.9) | <0.001 | | | |  |  |
| Revascularization, Coronary or Peripheral Arterial | 10635 (15.0) | | 4973 (15.5) | | | 5662 (14.5) | <0.001 | | | |  |  |
| *Other Cardiovascular Disease* |  |  | |  |  | | | | | | | |
| Atrial Fibrillation/Atrial Flutter | 19903 (28.0) | | 9862 (30.8) | | | 10041 (25.7) | | <0.001 | | | |  |
| Heart Failure | 27533 (38.8) | | 13986 (43.7) | | | 13547 (34.7) | | <0.001 | | | |  |
| Valvular Heart Disease | 15985 (22.5) | | 7512 (23.5) | | | 8473 (21.7) | | <0.001 | | | |  |
| Ventricular Arrhythmias | 5626 (7.9) | | 2756 (8.6) | | | 2870 (7.4) | | <0.001 | | | |  |
| *Systemic Autoimmune Disorders* |  | |  | | |  | |  | | | |  |
| Myositis | 149 (0.2) | | 58 (0.2) | | | 91 (0.2) | | 0.155 | | | |  |
| Multiple sclerosis | 286 (0.4) | | 143 (0.4) | | | 143 (0.4) | | 0.104 | | | |  |
| Rheumatoid Arthritis | 5584 (7.9) | | 2450 (7.7) | | | 3134 (8.0) | | 0.067 | | | |  |
| Seronegative Spondyloarthropathies | 2415 (3.4) | | 1045 (3.3) | | | 1370 (3.5) | | 0.077 | | | |  |
| Systemic Lupus Erythematosus | 475 (0.7) | | 180 (0.6) | | | 295 (0.8) | | 0.002 | | | |  |
| Other Systemic Connective Tissue Disorders | 478 (0.7) | | 201 (0.6) | | | 277 (0.7) | | 0.201 | | | |  |
| Vasculitis | 1508 (2.1) | | 696 (2.2) | | | 812 (2.1) | | 0.40 | | | |  |
| *Other Comorbidities* |  | |  | | |  | |  | | | |  |
| Asthma | 6809 (9.6) | | 3074 (9.6) | | | 3735 (9.6) | | 0.883 | | | |  |
| Chronic Obstructive Pulmonary Disease | 29305 (41.3) | | 14052 (43.9) | | | 15253 (39.1) | | <0.001 | | | |  |
| Diabetes Mellitus | 36847 (51.9) | | 17682 (55.3) | | | 19165 (49.1) | | <0.001 | | | |  |
| Hyperlipidemia | 61861 (87.1) | | 27726 (86.7) | | | 34135 (87.5) | | 0.001 | | | |  |
| Hypertension | 67874 (95.6) | | 30707 (96.0) | | | 37167 (95.3) | | <0.001 | | | |  |
| Hypothyroidism | 11443 (16.1) | | 5244 (16.4) | | | 6199 (15.9) | | 0.071 | | | |  |
| Liver Disease, Mild | 5853 (8.2) | | 2911 (9.1) | | | 2942 (7.5) | | <0.001 | | | |  |
| Liver Disease, Moderate or Severe | 1539 (2.2) | | 851 (2.7) | | | 688 (1.8) | | <0.001 | | | |  |
| Charlson comorbidity Index, median (IQR) | 4 (3-7) | | 5 (3-7) | | | 4 (2-6) | | <0.001 | | | |  |
| 1-2 | 15030 (21.2) | | 5780 (18.1) | | | 9250 (23.7) | | <0.001 | | | |  |
| 3-5 | 25985 (36.6) | | 11420 (35.7) | | | 14565 (37.3) | | <0.001 | | | |  |
| >5 | 29975 (42.2) | | 14784 (46.2) | | | 15191 (38.9) | | <0.001 | | | |  |
| Frailty | 19508 (27.5) | | 10414 (32.6) | | | 9094 (23.3) | | <0.001 | | | |  |
| Smoking Status, n (%) |  | |  | | |  | |  | | | |  |
| Current smoker | 15380 (21.7) | | 7120 (22.3) | | | 8260 (21.2) | | <0.001 | | | |  |
| Past smoker | 43640 (61.5) | | 19849 (62.1) | | | 23791 (61.0) | |  | | | |  |
| Never smoker | 10846 (15.3) | | 4606 (14.4) | | | 6240 (16.0) | |  | | | |  |
| Missing | 1124 (1.6) | | 409 (1.3) | | | 715 (1.8) | |  | | | |  |
| Medication Therapy, n (%) |  | |  | | |  | |  | | | |  |
| Angiotensin-Converting Enzyme Inhibitors/Angiotensin Receptor Blockers | 45619 (64.3) | | 20059 (62.7) | | | 25560 (65.5) | | <0.001 | | | |  |
| Anticoagulants | 15612 (22.0) | | 7654 (23.9) | | | 7958 (20.4) | | <0.001 | | | |  |
| Antihyperlipidemic | 52036 (73.3) | | 22963 (71.8) | | | 29073 (74.5) | | <0.001 | | | |  |
| Statin |  | |  | | |  | | <0.001 | | | |  |
| High Intensity | 23346 (32.9) | | 10522 (32.9) | | | 12824 (32.9) | |  | | | |  |
| Moderate Intensity | 25650 (36.1) | | 11134 (34.8) | | | 14516 (37.2) | |  | | | |  |
| Low Intensity | 2159 (3.0) | | 959 (3.0) | | | 1200 (3.1) | |  | | | |  |
| No Statin Use | 19835 (27.9) | | 9369 (29.3) | | | 10466 (26.8) | |  | | | |  |
| Antiplatelet | 43915 (61.9) | | 19703 (61.6) | | | 24212 (62.1) | | 0.202 | | | |  |
| Colchicine | 4381 (6.2) | | 2299 (7.2) | | | 2082 (5.3) | | <0.001 | | | |  |
| Glucagon-like Peptide-1 Receptor Agonists (GLP-1RA) | 1611 (2.3) | | 782 (2.4) | | | 829 (2.1) | | 0.005 | | | |  |
| Sodium-Glucose Transport Protein 2 Inhibitors (SGLT-2i) | 68724 (96.8) | | 30883 (96.6) | | | 37841 (97.0) | | 0.001 | | | |  |
| Systemic Corticosteroids | 22686 (32.0) | | 11207 (35.0) | | | 11479 (29.4) | | <0.001 | | | |  |
| Physiologic Measures, mean (SD) |  | |  | | |  | |  | | | |  |
| Body Mass Index, mean (SD) | 31.1 (6.5) | | 31.5 (7.0) | | | 30.9 (6.0) | | <0.001 | | | |  |
| Systolic Blood Pressure, mean (SD) | 135.2 (13.9) | | 135.1 (14.1) | | | 135.3 (13.7) | | 0.038 | | | |  |
| Diastolic Blood Pressure, mean (SD) | 74.0 (8.8) | | 73.8 (8.8) | | | 74.2 (8.8) | | <0.001 | | | |  |
| Laboratory Values, mean (SD) |  | |  | | |  | |  | | | |  |
| Hemoglobin A1c (Hb A1c) | 6.9 (1.5) | | 7.0 (1.5) | | | 6.8 (1.4) | | <0.001 | | | |  |
| eGFR (based on serum creatinine) | 48.2 (13.1) | | 47.2 (13.7) | | | 49.0 (12.6) | | <0.001 | | | |  |
| uACR (based on urine albumin) | 169.7 (471.2) | | 160.7 (472.6) | | | 178.6 (469.7) | | <0.001 | | | |  |
| LDL Cholesterol (LDL-C) | 88.4 (31.0) | | 87.6 (30.8) | | | 89.0 (31.1) | | <0.001 | | | |  |
| Region |  | |  | | |  | | <0.001 | | | |  |
| Northeast | 9463 (13.3) | | 4191 (13.1) | | | 5272 (13.5) |  | | |  |  |  |
| Midwest | 8036 (11.3) | | 3560 (11.1) | | | 4476 (11.5) |  | | |  |  |  |
| South | 30675 (43.2) | | 13665 (42.7) | | | 17010 (43.6) |  | | |  |  |  |
| West | 21166 (29.8) | | 9712 (30.4) | | | 11454 (29.4) |  | | |  |  |  |
| Non-Continental USA^*^ | 1650 (2.3) | | 856 (2.7) | | | 794 (2.0) |  | | |  |  |  |
| Abbreviation: hsCRP: high sensitivity c-reactive protein; SD: standard deviation; IQR: Interquartile range; CDC: Centers for Disease Control; SVI: Social vulnerability index; TIA: transient ischemic stroke; eGFR: estimated glomerular filtration rate; uACR: urine albumin creatinine ratio; LDL-C: low-density lipoprotein cholesterol  ^*^Alaska, Hawaii, Puerto Rico, US Virgin Islands | | | | | | | | |  |  |  |  |

# **Supplemental Table 5. Association Between hsCRP Quartiles and Adverse Cardiovascular Outcomes**

| **Outcomes** | **Model A** ^†^ | | | **Model B** ^‡^ | | |
| --- | --- | --- | --- | --- | --- | --- |
|  | hsCRP Quantile 2 (hsCRP>1.6 and <3.21) | hsCRP Quantile 3 (hsCRP>3.21 and <5.65) | hsCRP Quantile 4 (hsCRP>5.65 and <10) | hsCRP Quantile 2 (hsCRP>1.6 and <3.21) | hsCRP Quantile 3 (hsCRP>3.21 and <5.65) | hsCRP Quantile 4 (hsCRP>5.65 and <10) |
| 3-point MACE^§^ | 1.129* | 1.318* | 1.554* | 1.097* | 1.232* | 1.396* |
|  | (1.092 - 1.167) | (1.276 - 1.362) | (1.505 - 1.604) | (1.062 - 1.134) | (1.192 - 1.273) | (1.351 - 1.443) |
| 5-point MACE^‖^ | 1.135* | 1.294* | 1.468* | 1.106* | 1.226* | 1.350* |
|  | (1.101 - 1.169) | (1.257 - 1.333) | (1.426 - 1.512) | (1.073 - 1.139) | (1.190 - 1.262) | (1.311 - 1.391) |
| All-cause death | 1.125* | 1.330* | 1.606* | 1.093* | 1.238* | 1.432* |
|  | (1.087 - 1.165) | (1.286 - 1.376) | (1.553 - 1.660) | (1.056 - 1.132) | (1.197 - 1.282) | (1.384 - 1.482) |
| Cardiovascular death | 1.167* | 1.392* | 1.703* | 1.124* | 1.281* | 1.496* |
|  | (1.105 - 1.234) | (1.318 - 1.469) | (1.615 - 1.796) | (1.063 - 1.188) | (1.213 - 1.353) | (1.417 - 1.579) |
| Ischemic Stroke or TIA | 1.065 | 1.124 | 1.107 | 1.052 | 1.097 | 1.068 |
|  | (0.954 - 1.189) | (1.007 - 1.254) | (0.989 - 1.239) | (0.942 - 1.175) | (0.982 - 1.226) | (0.952 - 1.198) |
| Myocardial Infarction | 1.166* | 1.254* | 1.324* | 1.134* | 1.193* | 1.239* |
|  | (1.053 - 1.293) | (1.133 - 1.388) | (1.195 - 1.466) | (1.023 - 1.257) | (1.076 - 1.322) | (1.117 - 1.375) |
| Coronary revascularization | 1.101* | 1.174* | 1.144* | 1.077* | 1.146* | 1.113* |
|  | (1.036 - 1.169) | (1.106 - 1.246) | (1.077 - 1.215) | (1.014 - 1.144) | (1.079 - 1.217) | (1.047 - 1.184) |
| Peripheral arterial revascularization | 1.235* | 1.370* | 1.528* | 1.208* | 1.313* | 1.435* |
|  | (1.155 - 1.320) | (1.283 - 1.462) | (1.432 - 1.630) | (1.130 - 1.292) | (1.229 - 1.402) | (1.343 - 1.532) |

Abbreviations: ACD: All-cause death; AMI: Acute myocardial infarction; ASCVD: atherosclerotic cardiovascular disease; CVD: cardiovascular death; hsCRP: high-sensitivity C-reactive protein; MACE: Major adverse cardiovascular events; TIA: Transient Ischemic Attack

* Denotes p<0.05

| ^†^ Model A adjusted for age, sex, and year of hsCRP measurement. |
| --- |

^‡^ Model B adjusted for age, sex, year of hsCRP measurement, Charlson comorbidity index, prior MI, prior coronary revascularization, smoking status, body mass index, GFR, lipid-lowering therapies, antiplatelets, anticoagulants, angiotensin-converting enzyme inhibitors/angiotensin receptor blockers, SGLT2i, and GLP-1 RA

^§^ 3-point MACE includes all-cause death, MI, and ischemic stroke or TIA

^‖^ 5-point MACE includes all-cause death, MI, ischemic stroke or TIA, coronary revascularization, and peripheral artery revascularization

# **Supplement Table 6. Rates of Adverse Cardiovascular Outcomes with and without SI with Complete Case Analysis(with 95% Confidence Intervals)*^,^** ^†^

| **Cardiovascular Outcome** | **Hazard Ratio with SI** | |
| --- | --- | --- |
|  | **With Imputation** | **Complete Case Analysis**^‡^ |
| 3-point ASCVD MACE | 1.40* | 1.40* |
|  | (1.36 - 1.43) | (1.36 - 1.43) |
| 5-point ASCVD MACE | 1.41* | 1.41* |
|  | (1.38 - 1.44) | (1.38 - 1.44) |
| All-cause death | 1.19* | 1.19* |
|  | (1.16 - 1.22) | (1.16 - 1.22) |
| Cardiovascular death | 1.22* | 1.22* |
|  | (1.18 - 1.27) | (1.18 - 1.27) |
| Ischemic stroke or TIA | 1.30* | 1.30* |
|  | (1.19 - 1.43) | (1.19 - 1.43) |
| Myocardial  infarction | 1.18* | 1.18* |
|  | (1.09 – 1.28) | (1.09 – 1.28) |
| Coronary revascularization | 1.09* | 1.09* |
|  | (1.04-1.14) | (1.04-1.14) |
| Peripheral arterial revascularization | 1.24* | 1.24* |
|  | (1.18 - 1.31) | (1.18 - 1.31) |

Abbreviations: ASCVD: atherosclerotic cardiovascular disease; hsCRP: high-sensitivity C-reactive protein; MACE: major adverse cardiovascular outcomes; SI: systemic inflammation; TIA: transient ischemic attack.

^*^Indicates p<0.05 for hazard ratios evaluating association between SI (hsCRP>2mg/L and <10mg/L) and the outcome.

^†^ Adjusted for age, sex, year of hsCRP measurement, Charlson comorbidity index, prior MI, prior coronary revascularization, smoking status, body mass index, eGFR, lipid-lowering therapies, antiplatelets, anticoagulants, angiotensin-converting enzyme inhibitors/angiotensin receptor blockers, SGLT2i, and GLP-1 RA.

^‡^ Excludes individuals with missing body mass index or eGFR (n=1,221).

# **Supplemental Table 7. Rates of Adverse Cardiovascular Outcomes among Veterans with and without SI Excluding Individuals with Systemic Autoimmune Conditions**

| **Clinical Outcome** | **SI (hsCRP>2 and <10)** (N=33,503) | **Without SI (hsCRP<2)** (N=20,087) | **p-value for difference** |
| --- | --- | --- | --- |
| 3-point ASCVD MACE^*^ | 11.79 | 8.93 | <0.001 |
|  | (11.63 - 11.96) | (8.74 - 9.11) |  |
| 5-point ASCVD MACE^†^ | 15.53 | 11.43 | <0.001 |
|  | (15.32 - 15.73) | (11.21 - 11.65) |  |
| All-cause death | 10.66 | 9.40 | <0.001 |
|  | (10.51- 10.82) | (9.22 - 9.59) |  |
| Cardiovascular death | 4.27 | 3.58 | <0.001 |
|  | (4.17 - 4.38) | (3.46 - 3.7) |  |
| Ischemic stroke or TIA | 0.80 | 0.62 | 0.002 |
|  | (0.76 - 0.85) | (0.57 - 0.67) |  |
| Myocardial infarction | 0.99 | 0.82 | <0.001 |
|  | (0.94 - 1.04) | (0.77 - 0.88) |  |
| Coronary revascularization | 3.11 | 2.33 | <0.001 |
|  | (3.02 - 3.2) | (2.24 - 2.43) |  |
| Peripheral arterial revascularization | 2.80 | 2.11 | <0.001 |
|  | (2.72 - 2.89) | (2.02 - 2.2) |  |

Abbreviations: ACD: All-cause death; AMI: Acute myocardial infarction; ASCVD: atherosclerotic cardiovascular disease; CVD: cardiovascular death; hsCRP: high-sensitivity C-reactive; TIA: Transient Ischemic Attack.

| * 3-point MACE includes all-cause death, MI, and ischemic stroke or TIA  ^†^ 5-point MACE includes all-cause death, MI, ischemic stroke or TIA, coronary revascularization, and peripheral artery revascularization |
| --- |
|  |

# **Supplemental Table 8. Rates of Adverse Cardiovascular Outcomes with and without SI Including hsCRP>10mg/L**

| **Clinical Outcome** | **With SI (hsCRP>2) (N=70,990)** | **Without SI (hsCRP<2) (N=23,176)** | **p-value** |
| --- | --- | --- | --- |
|  |  |  |  |
| 3-point ASCVD MACE^*^ | 12.43 | 8.66 | <0.001 |
|  | (12.3 - 12.55) | (8.49 - 8.83) |  |
| 5-point ASCVD MACE^†^ | 15.73 | 11.07 | <0.001 |
|  | (15.57 - 15.87) | (10.87 - 11.27) |  |
| All-cause death | 12.73 | 9.31 | <0.001 |
|  | (12.6 - 12.85) | (9.14 - 9.48) |  |
| Cardiovascular death | 4.95 | 3.50 | <0.001 |
|  | (4.87 - 5.03) | (3.39 - 3.61) |  |
| Ischemic stroke or TIA | 0.74 | 0.61 | 0.002 |
|  | (0.71 - 0.77) | (0.57 - 0.66) |  |
| Myocardial infarction | 0.98 | 0.81 | <0.001 |
|  | (0.94 - 1.01) | (0.76 - 0.86) |  |
| Coronary revascularization | 2.71 | 2.29 | <0.001 |
|  | (2.95 - 3.07) | (2.2 - 2.37) |  |
| Peripheral arterial revascularization | 3.01 | 2.07 | <0.001 |
|  | (2.95 - 3.07) | (1.98 - 2.15) |  |

Abbreviations: ACD: All-cause death; AMI: Acute myocardial infarction; ASCVD: atherosclerotic cardiovascular disease; CVD: cardiovascular death; hsCRP: high-sensitivity C-reactive protein; MACE: Major adverse cardiovascular events

* 3-point MACE includes all-cause death, MI, and ischemic stroke or TIA

^†^ 5-point MACE includes all-cause death, MI, ischemic stroke or TIA, coronary revascularization, and peripheral artery revascularization

# **Supplement Table 9. Annual Rates of Acute Care Utilization among Veterans with and without SI**

| **Outcomes** | **Overall**  **(N=69,103)** | **hsCRP>2 and <10 (N=48,224)** | **hsCRP<2 (N=20,879)** | **p-value*** |
| --- | --- | --- | --- | --- |
| All Cause Hospitalizations | 0.76 (0.75 - 0.76) | 0.79 (0.79-0.79) | 0.70 (0.69-0.70) | <.0001 |
| Cardiovascular Hospitalizations | 0.20 (0.20-0.20) | 0.21 (0.21-0.21) | 0.19 (0.18-0.19) | <.0001 |
| Emergency Department Visits | 1.46 (1.45-1.45) | 1.47 ((1.46 - 1.47) | 1.43 (1.42-1.44) | 0.007 |

Abbreviations: hsCRP: high-sensitivity C-reactive protein

^*^Indicates p<0.05 for incidence rate ratio between SI (hsCRP>2mg/L and <2mg/L) and the outcome.

#

# **Supplement Figure 1. Histogram of hsCRP Levels**

**
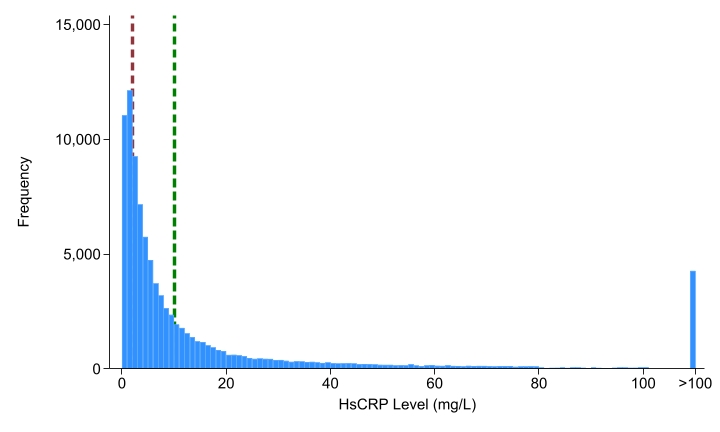
**

The red line with short dashes represents the cut-off of 2mg/L. The green line with long dashes represents the cutoff of 10mg/L.

# **Supplement Figure 2. Association Between hsCRP Modeled as a Spline and 3-point MACE**


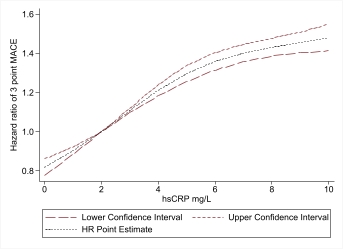


Abbreviations: HR: hazard ratio; hsCRP: high-sensitivity C-reactive protein; LCI: Lower 95% Confidence Interval; MACE: Major adverse cardiovascular events; UCI: Upper 95% Confidence Interval.
